# Light-Based 3D Printing of Polyesters: From Synthesis to Fabrication

**DOI:** 10.1021/acs.chemrev.5c00611

**Published:** 2025-12-23

**Authors:** Quinten Thijssen, Astrid Quaak, Bart Bijleveld, Bo Li, Lenny Van Daele, Andreas Heise, Sandra Van Vlierberghe

**Affiliations:** † Polymer Chemistry and Biomaterials Group, Centre of Macromolecular Chemistry, Department of Organic and Macromolecular Chemistry, 98721 Ghent University, Krijgslaan 291, 9000 Gent, Oost-Vlaanderen, Belgium; ‡ Department of Chemistry, RCSI University of Medicine and Health Sciences, Dublin D02 YN77, Ireland; § CURAM the SFI Research Centre for Medical Devices, RCSI University of Medicine and Health Sciences, Dublin D02 YN77, Ireland; ∥ The SFI Centre for Advanced Materials and BioEngineering Research, RCSI University of Medicine and Health Sciences, Dublin D02 YN77, Ireland

## Abstract

Polyesters represent a versatile class of materials whose biodegradability, biocompatibility, mechanical tunability, and broad chemical design space have made them valuable across a wide range of application areas, including tissue engineering, biomedical engineering, sustainable manufacturing, and soft robotics. Light-based 3D printing has further expanded their potential by enabling precise spatial control across nano- to macroscales, supporting the fabrication of resorbable implants, drug-delivery systems, microneedle arrays, and stimuli-responsive materials. This review discusses the essential steps toward light-based 3D printing of polyesters from synthetic strategies for producing these materials to functionalization methods that render them suitable for light-based 3D printing. Particular attention is given to the synthetic origin of the polyester, the way photoreactive groups are introduced and organized within the network, and how the formulation of the resulting photoresin together govern the ultimate photoreactivity, degradation behavior, print resolution, and mechanical performance. Advantages and limitations of current photochemical approaches are discussed across different light-based 3D printing technologies. With continuing advancements in manufacturing, the field of light-based 3D printing of polyesters shows substantial promise, poised to redefine material design, and influence a broad range of future technologies.

## Graphical abstract

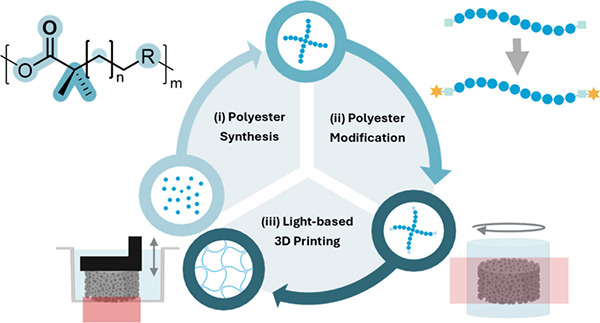

## 1 Introduction

Polyesters represent a remarkably versatile class of polymers whose behavior spans a broad continuum, from amorphous to semicrystalline, from rapidly to slowly degrading, from transparent to opaque, and from hard to soft and highly ductile.^1–4^ This breath of behavior has made polyesters central not only to tissue engineering and regenerative medicine but also to emerging directions in recyclable materials, drug delivery, soft robotics, stimuli responsive, and programmable materials.

At the molecular level, the defining ester moiety plays a central role in dictating the macroscopic behavior of the 3D printed polyester.^5^ Its permanent dipole moment imparts polarity; the electrophilic carbonyl carbon renders the backbone susceptible to hydrolysis, and its planar, sp^2^-hybridized geometry introduces conformational rigidity. These features govern hydrolytic stability, crystallization tendencies, and mechanical performance.^6,7^ Beyond the ester group itself, a broad set of additional structural motifs can be introduced to tailor the material’s performance, including variations in alkyl chain length, the incorporation of functional groups such as unsaturated double bonds (including their position relative to the ester), stereochemistry and tacticity of the polymer backbone, the presence of aromatic moieties, and the degree of branching.^8,9^

The introduction of light-triggered reactivity further expands this design space. When polyesters are endowed with photo-cross-linkable groups, their structural and physicochemical characteristics become directly coupled to the mechanisms and kinetics of photopolymerization.^10–12^ This coupling presents both opportunity and complexity: the same features that give polyesters their mechanical and degradative tunability, molar mass, architecture, crystallization behavior, and segmental mobility, also determine whether they can be formulated into resins that cure rapidly, uniformly, and with high fidelity under layerwise, nonlinear, or volumetric 3D printing conditions. As a result, rational design of polyester-based photoresins requires an integrated understanding of synthesis, functionalization, network formation, and resin-level formulation.^3,4,13–15^

Realizing robust 3D constructs from polyesters, therefore, entails three interconnected stages (Figure 1). First, polymerization mechanisms define the architectures, molar masses, and sequence features that establish the structural and physicochemical boundaries within which printable polyesters must reside (section 2). Second, functionalization chemistry determines how photo-cross-linkable groups are introduced and how the chosen photochemical mechanism—radical chain-growth, radical step-growth, or nonradical—controls kinetics, gelation behavior, network topology and homogeneity, oxygen sensitivity, and shrinkage (section 3). Third, resin formulation governs how these precursors perform under the constraints imposed by specific printing modalitie, as well as how the distinct stages combine to determine the final properties of the light-based 3D-printed polyester (xection [Sec sec4]).

**Figure 1:**
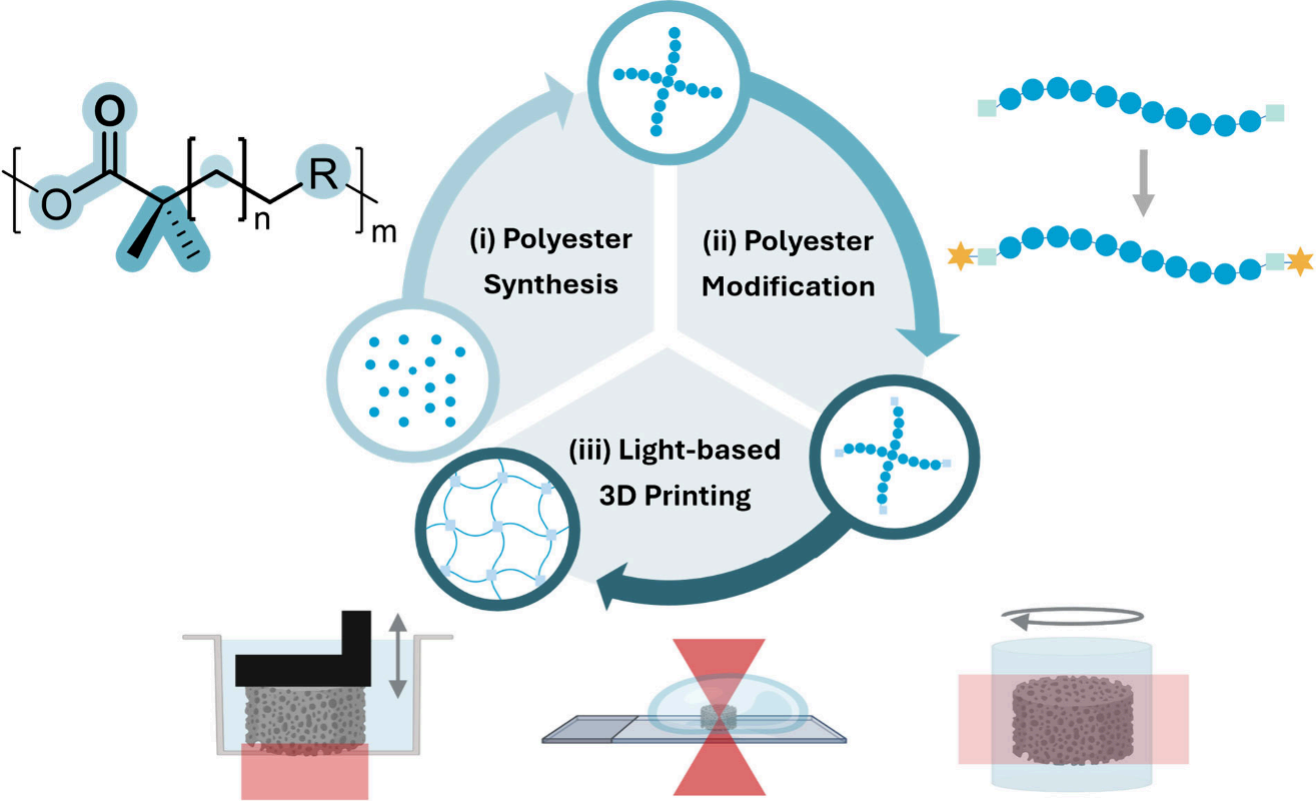
Schematic overview of the review structure, which is organized around three key stages required to translate polyesters into functional 3D constructs via light-based 3D printing: (i) Polyester synthesis, highlighting how structural diversity arises from monomer selection and polymerization strategies. A general polyester structure is shown to illustrate this chemical versatility, including variation in alkyl chain length, incorporation of functional groups or aromatic moieties (e.g., unsaturated double bonds and their position relative to the ester, denoted as R), stereochemistry and tacticity of the polymer backbone, and degree of branching. (ii) Chemical modification of polyesters to introduce photo-cross-linkable functionalities, enabling network formation upon light exposure. (iii) Light-based 3D printing of photo-cross-linkable polyesters. Together, these sections outline a modular framework for designing polyester-based materials for light-based 3D printing.

By integrating these three layers, synthetic design, photoreactive functionalization, and formulation for light-based 3D printing, this review provides a chemical framework for translating the structural tunability of polyesters into application-relevant and functional 3D architectures. The discussion highlights both the opportunities and the limitations inherent to polyester-based systems, offering guidance for designing next-generation resins that combine print fidelity, mechanical robustness, controlled degradability, and functional versatility across a broad application landscape.

## 2 From Monomer to Polyester

Polyester synthesis determines the chemical landscape long before any photochemistry is introduced. The monomer set, the polymerization mechanism, and the placement of the reactive sites during synthesis collectively define the windows within which all subsequent formulation and curing steps must operate. This section therefore maps the accessible polyester space, what backbone chemistries can be made, which architectures they yield, and which intrinsic property regimes they occupy. The emphasis is on structural consequences rather than on synthetic detail. For a more in-depth discussion of synthetic strategies, the reader is referred to the available literature.^16–19^ The following subsection (section 2.2) examines how the features imposed at this synthetic stage, chain end identity, chain-length distributions, and architectural constraints, set the boundary conditions for converting these polyesters into photoreactive precursors suitable for light-based 3D printing.

### 2.1 Synthetic Routes to Polyester Backbones

The structural diversity of polyester backbones is determined first and foremost by the polymerization mechanism through which they are constructed. Although the field encompasses decades of development across organometallic, organocatalytic, enzymatic, and radical methodologies, four synthetic families dominate contemporary routes to well-defined polyesters: ring-opening polymerization (ROP), polycondensation, ring-opening copolymerization (ROCOP), and radical ring-opening polymerization (rROP).^1,8,16,18,20–25^ Alongside these synthetic platforms, biologically derived polyhydroxyalkanoates (PHAs) constitute an increasingly relevant class of polyesters and are discussed separately below.

#### 2.1.1 Ring-Opening Polymerization

Ring-opening polymerization (ROP) remains the most versatile and widely adopted route to well-defined aliphatic polyesters. Its appeal stems from the combination of broad monomer scope, predictable chain-growth behavior, and the ability to regulate chain ends, molar mass, dispersity, and architecture with a degree of precision that is unmatched by other polyester-forming reactions. The family of monomers accessible by ROP spans strained lactones and lactides, higher-ring macrocycles, and even heterocyclic monomers such as morpholine-2,5-diones, enabling access to polyesters, poly­(ester–amide)­s, and copolyesters with finely tunable intrinsic properties.^23,26–29^

Classical ROP monomers, including ε-caprolactone, δ-valerolactone, lactide, and glycolide, cover a wide spectrum of crystallinity, hydrophobicity, and degradation kinetics.^30–32^ ε-Caprolactone yields semicrystalline, slowly hydrolyzing polyesters; lactide and glycolide enable higher *T*_g_/*T*_m_ ranges and substantially faster hydrolysis. δ-Valerolactone occupies an intermediate regime, producing polyesters with moderate crystallinity and degradation rates that bridge the gap between ε-caprolactone and the more rapidly hydrolyzing lactone systems. Further expanding the monomer scope to less strained or more conformationally flexible rings significantly broadens this landscape: γ-butyrolactone (γ-BL), traditionally considered “non-polymerizable,” can undergo ROP under specific low-temperature organocatalytic conditions;^33,34^ very large ω-lactones give access to highly flexible, low-*T*_g_ polyesters; and morpholine-2,5-diones introduce amide linkages, increasing intermolecular interactions and elevating stiffness and *T*_g_.^23,26,27^ Across this monomer space, backbone architecture, and thereby access to soft, hard, semicrystalline, or glassy regimes, can be tuned with high precision.

ROP also supports both block and statistical copolymer formation.^35^ Sequential monomer addition enables the synthesis of block copolyesters when the first monomer is nearly fully consumed before introducing the second, whereas cofeeding monomers with similar reactivity ratios yields random or gradient copolymers (Figure 2). These design choices dramatically expand the accessible property space: crystallinity can be disrupted or reinforced, hydrophobic/hydrophilic balances modulated, and degradation kinetics tuned across orders of magnitude.

**Figure 2:**
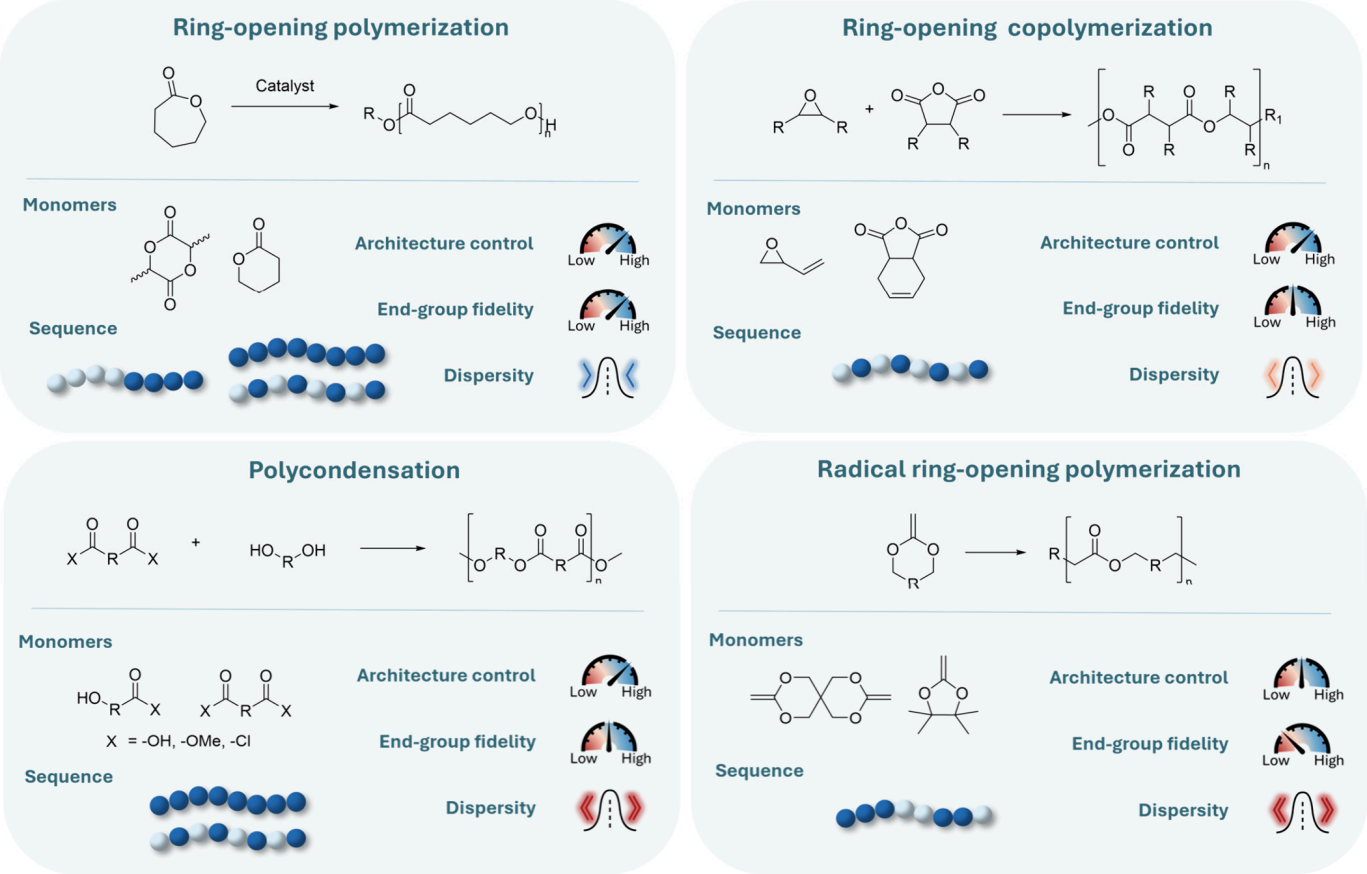
Mechanistic families for polyester backbone formation and their structural consequences. (top left) Ring-opening polymerization (ROP) of cyclic esters; (top right) ring-opening copolymerization (ROCOP) of epoxides with cyclic anhydrides; (bottom left) polycondensation of diacids (or derivatives) with diols; (bottom right) radical ring-opening polymerization (rROP) of cyclic ketene acetals. For each mechanism, representative monomers, the resulting sequence regularity, and qualitative trends in architecture control, end-group fidelity, and dispersity are indicated. Gauges and distribution sketches provide qualitative rather than quantitative comparisons and link directly to the functional-group and molecular-weight considerations discussed in section 2.2.

As polymerization proceeds, suppression of transesterification is critical: backbiting, chain scrambling, and intramolecular cyclization broaden dispersity and obscure chain end fidelity.^1,36–40^ The classical benchmark for lactone ROP is tin­(II) octanoate, a coordination–insertion catalyst capable of producing well-defined polyesters at scale. However, it typically requires elevated temperatures (≥100 °C) and extended reaction times, and its metal residue profile remains a consideration in some applications.^41–43^ Organometallic alternatives, including aluminum alkoxides, aluminum salen complexes, and zinc or magnesium catalysts, offer tunable reactivity, narrower dispersities, and compatibility with various monomers, often operating at lower temperatures and providing cleaner end-group profiles.^21,22,43^

Organocatalysts have substantially expanded the synthetic space. Thiourea-based catalysts, introduced by Lohmeijer and co-workers, enable hydrogen-bond-assisted activation of the monomer and initiator, suppressing transesterification and yielding polyesters with impressively narrow dispersities.^36^ In some systems, polymerization proceeds at room temperature and can reach high conversion within seconds to minutes, a dramatic contrast to the hours required under classical tin catalysis. Strong organic bases (e.g., triazabicyclodecene (TBD), 1,8-diazabicycloundec-7-ene (DBU), phosphazenes), guanidines, and *N*-heterocyclic carbenes also promote ROP through nucleophilic activation, although their high basicity requires careful control to avoid side reactions or chain scission.^44^ Each catalyst class defines a distinct operational envelope, temperature, monomer compatibility, solvent requirements, and reaction kinetics, making catalyst choice a primary determinant of accessible polymer structures.^16,45–47^

Beyond coordination–insertion and organocatalytic strategies, three additional ROP modalities occupy important space in polyester synthesis: anionic, cationic, and enzymatic ROP. Anionic ROP employs nucleophilic initiators such as alkoxides or carbanions and enables rapid polymerization of electrophilic cyclic esters, but the charged propagating species are highly susceptible to transesterification and backbiting, which complicates control over molar mass and dispersity. Cationic ROP proceeds through electrophilic activation of the monomer, typically by Brønsted or Lewis acids, to generate oxonium intermediates. This route is valuable for electron-rich or highly strained monomers, yet propagation is sensitive to impurities and often operates through mixed active-chain end and active-monomer pathways, limiting architectural precision.^1,45,48,49^ Enzymatic ROP, mediated by lipases or esterases, provides access to polyesters under exceptionally mild conditions and offers high monomer selectivity, particularly for large macrocyclic lactones.^49–54^ However, its activated-monomer mechanism generally precludes deterministic control over chain end identity and molar mass.^49^ Together, these ionic and enzymatic routes broaden the monomer scope and processing conditions, but their intrinsic mechanistic constraints limit the chain end fidelity and dispersity control that underpin most well-defined photoreactive polyester precursors discussed in section 2.2.

ROP therefore represents the most established and chemically flexible route to aliphatic polyesters. Its strengths lie in its architectural precision, monomer breadth, and control over chain end identity and molar mass. Its limitations, most notably, sensitivity to transesterification, catalyst-removal considerations, and the high temperatures required by some catalyst systems, are well recognized but continue to be addressed through advances in catalyst design. In the context of this review, ROP defines the structural starting point from which most photoreactive polyester precursors originate, setting the architectural and compositional boundaries examined further in section 2.2.

#### 2.1.2 Polycondensation

Polycondensation represents the classical and industrial backbone-forming route to polyesters, particularly when high-melting, (semi)­aromatic structures are required. In contrast to the chain-growth nature of ROP, polycondensation proceeds through a step-growth mechanism in which bifunctional monomers, typically diols and diacids, diesters, or acid chlorides, react with release of small molecules such as water, methanol, or hydrogen chloride (Figure 2).^55^

The monomer space accessible through polycondensation is broad. Aliphatic diacids (e.g., succinic, adipic) combined with diols (e.g., ethylene glycol, 1,4-butanediol) yield materials such as poly­(butylene succinate) or poly­(butylene adipate), whereas terephthalic or isophthalic acids paired with ethylene glycol give rise to industrial workhorses such as poly­(ethylene terephthalate) (PET) and poly­(butylene terephthalate) (PBT).^56–59^ These aromatic and semiaromatic polyesters are notable for their high crystallinity, elevated *T*_g_ and *T*_m_, and slow hydrolytic degradation—features fundamentally linked to the hydrophobic nature, rigidity, and packing efficiency of the incorporated aromatic units.^60–62^ Introduction of multifunctional monomers such as glycerol or citric acid allows controlled branching or network precursors, exemplified by poly­(glycerol sebacate) (PGS), which occupies a contrasting regime of elastomeric, low-*T*_g_ polyesters.^63,64^

Catalyst systems span traditional antimony oxides, titanium and tin derivatives, and zinc salts, chosen for their ability to accelerate esterification or transesterification under melt conditions. More recent organocatalytic and enzymatic approaches offer milder alternatives but remain far less established, especially for producing high-molar mass materials at scale.^21,29,65–68^ Regardless of the catalyst family, polycondensation generally relies on high temperatures and long reaction times, reflecting the need to overcome the equilibrium limitations and diffusion constraints in viscous melts.

Whereas ROP excels at architectural precision and clean end-group definition, polycondensation defines a complementary region of the polyester space dominated by high-performance materials and step-growth-derived architectures. The next section addresses ring-opening copolymerization, a polymerization that provides access to alternating polyesters.^21,64,69^

#### 2.1.3 Ring-Opening Copolymerization

Ring-opening copolymerization (ROCOP) represents an alternative chain-growth route to polyesters that closely parallels ring-opening polymerization (ROP) but significantly broadens the accessible monomer space by coupling epoxides with cyclic anhydrides under precise catalyst control.^70,71^

At the mechanistic level, ROCOP operates through catalyst-mediated activation of an initiator (usually a hydroxyl-bearing initiator) followed by nucleophilic attack on either the epoxide or anhydride, resulting in the formation of an alkoxide or carboxylate intermediate that subsequently reacts with the complementary monomer (Figure 2).^72,73^ This catalytic cycle enforces strict alternation between the two monomers at the propagation chain end, while minimizing epoxide–epoxide coupling and other undesired side reactions.^70,74–76^

Metal–salen complexes (Cr, Co, Al) remain among the most widely investigated catalysts across diverse monomer combinations.^72,73^ Lewis pair catalysts, such as triethylborane paired with DBU or TBD, operate through cooperative activation of the epoxide and stabilization of the propagating alkoxide, affording high selectivity for alternating insertion. *N*-Heterocyclic carbenes and bifunctional organocatalysts have also proven effectiveness in certain epoxide-anhydride combinations, though often with narrower monomer compatibility.^76–79^

ROCOP’s principal strength lies in its monomer breadth and functional tolerance. Epoxides include simple oxiranes (ethylene oxide, propylene oxide), glycidyl ethers, functionalized epoxides, and larger substituted cyclic ethers, while anhydrides span aliphatic, aromatic, and rigid bicyclic anhydrides.^9,71^ Many of these monomers contain polar, asymmetric, or sterically demanding features. ROCOP therefore provides access to polyesters whose repeating units incorporate polar substituents, rigid aromatic fragments, or pendant functional groups directly incorporated from the monomer design.

Despite these advantages, ROCOP remains less (industrially) established than ROP or polycondensation. Many catalytic systems are sensitive to impurities or moisture; several require costly or air-sensitive complexes, and large-scale protocols are less standardized. Nonetheless, ROCOP offers a strategically important extension of polyester chemistry as it provides polyester architectures that cannot be readily achieved through other polymerization routes.^70,72,76^

Whereas ROCOP extends polyester formation to epoxide/anhydride systems, radical ring-opening polymerization expands the design space further by enabling the radical-mediated polymerization of polyesters.

#### 2.1.4 Radical Ring-Opening Polymerization

Radical ring-opening polymerization (rROP) occupies a unique conceptual space at the interface of vinyl polymerization and polyester chemistry. Through the use of cyclic ketene acetals (CKAs) and related monomers, rROP enables the radical-mediated polymerization of polyesters, thereby providing a radical pathway toward degradable polymers (Figure 2).^8,18,80^

The mechanistic core of the rROP is the competition between ring-opening and ring-retaining propagation pathways. In a typical CKA, radical addition occurs at the exocyclic C = C bond to generate a transient radical intermediate. This intermediate may undergo:

1. Ring-opening, cleaving the C–O bond and producing a propagating radical that carries an ester linkage in the chain, or
2. Ring-retaining propagation, in which the radical adds to another monomer without opening the ring, yielding a conventional vinyl repeat unit.

The ratio of these two pathways dictates the polyester content and thus the degradability of the resulting backbone. Achieving high ring-opening selectivity is therefore the central synthetic challenge of rROP.^80,81^

The monomer scope of rROP is dominated by CKAs such as 2-methylene-1,3-dioxolane, 2-methylene-1,3-dioxepane, and benzo-fused analogues.^18,80–82^ These monomers vary in ring size and substitution pattern, both of which influence ring strain and the stability of the intermediate radicals. Larger rings and benzo-annulated systems often bias the reaction toward ring-opening by stabilizing the developing radical and lowering the energy barrier for C–O cleavage. rROP is frequently conducted as a copolymerization with classical vinyl monomers, acrylates, methacrylates, styrenics, to tune *T*_g_, modulus, processability, and hydrophobicity.^82–84^ In such cases, the CKA typically behaves as the minor comonomer, and the challenge becomes achieving meaningful ester incorporation without sacrificing the propagation rate or introducing uncontrolled sequence defects.

Despite these challenges, rROP remains an intellectually compelling strategy for merging radical polymerization versatility with polyester-like degradability, offering access to materials not achievable by other routes. Its conceptual value has inspired extensive mechanistic investigation, although its practical deployment is still limited, relative to the maturation of ROP, ROCOP, and polycondensation. Continued monomer development and improved control over ring-opening selectivity will determine whether rROP can evolve into a broadly applicable platform for constructing degradable (co)­polymer architectures.^80–82,85^

The final synthetic family considered here is biological in origin: polyhydroxyalkanoates (PHAs), produced via microbial metabolic pathways, which provide an orthogonal access route to polyester backbones.^86–88^

#### 2.1.5 Biological Polyester Production: Polyhydroxyalkanoates (PHAs)

Polyhydroxyalkanoates (PHAs) constitute a class of polyesters synthesized intracellularly by bacteria. Enzymatic polymerization of hydroxyacyl-CoA intermediates yields highly stereoregular chains that accumulate as intracellular granules. Upon cell lysis and polymer recovery, these granules often display semicrystalline character, although the crystalline state depends strongly on extraction and purification conditions (e.g., solvent choice, temperature, precipitation method).^86^ Two families dominate the PHA landscape. Short-chain-length PHAs (scl-PHAs), such as poly­(3-hydroxybutyrate) (P3HB) and its 3-hydroxyvalerate copolymers, are semicrystalline, stiff, and slowly degrading due to their high stereoregularity. Medium-chain-length PHAs (mcl-PHAs) show lower crystallinity, low *T*_g_, and elastomeric behavior, expanding accessible properties toward soft and flexible materials. In both cases, crystallinity, melting temperature, and degradation behavior are strongly influenced by the regularity of the biosynthetic repeat units and by the specific biosynthetic and processing conditions.^87,88^

PHAs therefore form a biologically derived complement to synthetic polyester families: stereoregular and renewable at the repeat-unit level, but constrained by limited end-group control, broader dispersities, and narrow thermal processing windows.^88,89^ These characteristics place PHAs within the broader polyester landscape considered in section 2.2, where chain end identity, molar mass distributions, backbone regularity, and crystallization behavior are evaluated in terms of their relevance for converting polyesters—whether synthetic or biogenic—into photoreactive precursors suitable for light-based 3D printing.

### 2.2 Polyester Synthesis Considerations for Light-Based Polyester 3D Printing

The suitability of a polyester for light-based 3D printing is fixed at the moment that its backbone is constructed. The polymerization mechanism determines which functional groups are present at the chain ends, how regular these termini are across the polymer population, and which additional functional groups were introduced during the polymerization. These synthesis-imposed features govern whether photoreactive units can be installed in a controlled fashion, how many can be introduced, and how uniformly they can contribute to network formation. Equally important, they dictate how much of the original polyester behavior, crystallinity, flexibility, thermal response, and degradation pattern, can be preserved once a cross-linked network is formed.

This subsection therefore examines the mechanistic origins of the structural features inherited from synthesis: functional-group identity, chain end fidelity, options for backbone-level handle incorporation, and the attainable molar mass and dispersity windows. Together, these features define the structural boundaries within which polyesters can later be converted into photo-cross-linkable precursors.

#### 2.2.1 Functional-Group Definition Emerging from Polymerization Mechanism

Polymerization mechanisms differ fundamentally in whether they provide intrinsically reactive sites for downstream functionalization. When the mechanism produces well-defined, uniform end-groups, these termini constitute a direct and quantitative route to installing photoreactive units. When it does not, functionalization must rely on externally introduced reactive groups (section 2.2.2).

ROP provides programmable and quantitatively addressable termini. In ROP, each initiated chain grows uniformly, and the identity of the α-terminal group is fixed by the initiator structure while the ω-terminal group is defined by the propagating chain end (Figure 3).^90,91^ Suppression of transesterification preserves this fidelity throughout polymerization, enabling quantitative installation of photoreactive units and predictable α,ω-telechelic or multiarm architectures. In addition, the choice and functionality of the initiator determine how many termini each chain carries and what chemical identity they possess, a feature that can later be exploited to introduce specific functional units at the α-chain end (section 3.1.2.2).^1,36,40,75^ This determinism in ROP explains why most photo-cross-linkable polyesters originate from this family: every chain carries the same number and identity of reactive sites, providing a direct and reliable route to well-defined precursor topologies (Figure 3). It should be noted, however, that enzymatic ROP follows an activated-monomer mechanism in which chain initiation and termination are not externally controlled. Terminus identity emerges from enzyme–substrate interactions and can shift with monomer structure, temperature, or impurities, providing limited reproducibility.^92–94^

**Figure 3:**
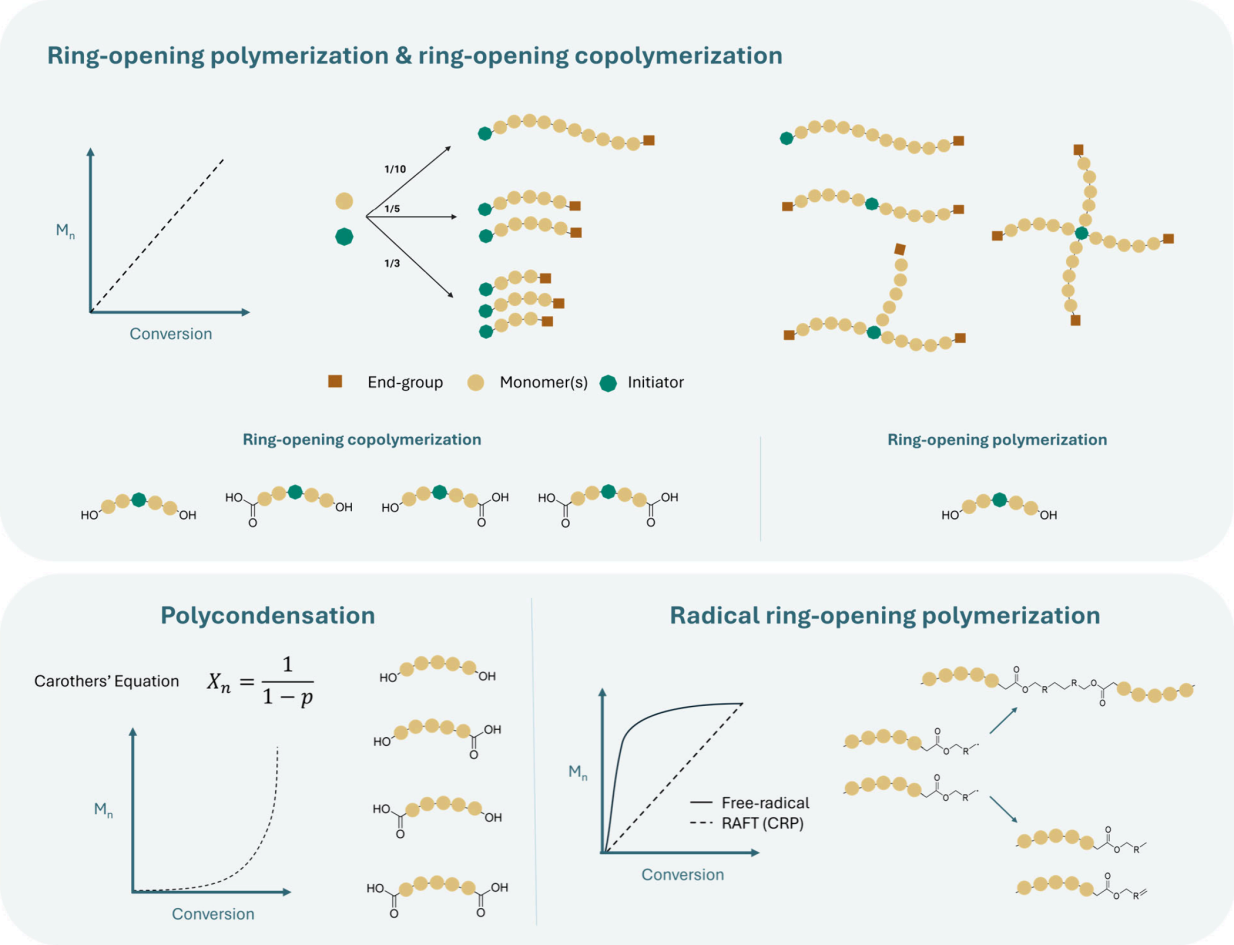
Comparison of polyester-forming mechanisms and their consequences for end-group fidelity, *M*_n_-conversion progression, and polymer architecture. ROP provides living or pseudoliving growth with uniform α,ω-termini, narrow dispersities, and well-defined architectures. ROCOP yields alternating epoxide–anhydride backbones with controlled molar mass and architecture; end-groups can be biased toward hydroxyl termini by using an epoxide excess, and *M*_n_ increases approximately linearly with conversion. Polycondensation generates inherently statistical end-groups, and *M*_n_ increases only at high conversion according to Carothers’ relation. Radical ring-opening polymerization (rROP) produces uncontrolled termini via radical termination (recombination and disproportionation) and a rapid early rise in *M*_n_; coupling to controlled radical polymerization (CRP) methods (e.g., RAFT) can impose pseudoliving *M*_n_-conversion behavior.

In ROCOP, the polymer chain grows through the alternating incorporation of two different monomers, which inherently increases the structural complexity of the resulting polyester precursor (Figure 3). Despite this alternation, the process follows a living or pseudoliving chain-growth mechanism similar to that observed in ring-opening polymerization (ROP), allowing control over molar mass and dispersity.^70,75^ However, achieving well-defined end-group functionalization requires careful control over the monomer feed. In practice, a slight excess of epoxide is typically introduced to bias the final insertion step, thereby ensuring hydroxyl-terminated chains rather than carboxylate-terminated ones.^71,74,75^

Polycondensation follows a classical step-growth polymerization mechanism according to Carothers’ equation. As a consequence, since the end-groups are determined statistically rather than by the presence of an initiator, the polymer population typically contains mixtures of hydroxyl, and carboxyl acid groups (Figure 3).^21,29,67^ Theoretically, stoichiometric bias (e.g., diol excess) can shift the population toward one end-group type; however, this remains to be experimentally verified as a viable route to controlled photo-cross-linkable precursors. Dual-pathway strategies—functionalizing both termini independently—are conceptually possible but add synthetic steps and have not yet been reported.^9,70^

The remaining polyester-forming strategies share a common outcome, poor end-group fidelity, but for mechanistically distinct reasons:

- **rROP** produces termini through radical termination and transfer. The identity of the chain end depends on the specific termination pathway (combination, disproportionation) and on the competition between ring-opening and ring-retaining propagation. As a result, neither the chemical identity nor the number of chain ends can be programmed (Figure 3).^18,81^

- **PHAs** inherit their termini entirely from metabolic polymerization and extraction chemistry. Chain ends reflect enzyme-controlled initiation and termination steps inside the cell and are further altered by conditions used for polymer recovery. End-group definition is therefore inherently stochastic.^88,89^

For all routes, functionalization must rely on backbone-embedded reactive groups (biosynthetic, monomer-derived, or introduced statistically). Quantitative end-group modification is rarely feasible, and the structural heterogeneity introduced at the synthesis propagates into functionalization, resin formulation, and printing.

#### 2.2.2 End-Group vs In-Chain Incorporation

When the polymerization mechanism provides uniform, addressable end-groups, those termini constitute the most straightforward reactive groups for later modification. In such architectures, the polyester segment between the α- and ω-termini remains uninterrupted and any subsequent functionalization can, in principle, be confined to the chain ends. The resulting network then links long, chemically homogeneous polyester strands, and the molar mass of the precursor maps directly onto the molar mass between cross-links when end-functionalization is quantitative.^92,93,95^

When end-group fidelity is low, or when a higher density of reactive groups is required, functionalization must shift from termini to the backbone. In this case, reactive groups arise either from comonomers that are built into the chain during polymerization or from postpolymerization transformations that convert internal units into reactive sites. A central structural consequence is that each backbone handle interrupts the continuous polyester sequence, partitioning the chain into shorter subsequences that are later connected into the network. As the density of such internal reactive sites increases, the polyester backbone becomes progressively segmented, and the network is constructed from many shorter polyester segments rather than a small number of long strands.^9,70^

This change in the handle location has two direct implications for light-based 3D printing. First, it broadens the effective distribution of strand lengths in the network because the distances between reactive groups now reflect sequence statistics rather than a single, well-defined chain length. Second, it makes it more difficult to preserve the intrinsic behavior of the parent polyester, crystallization, thermal transitions, degradation profile, because the polyester motif is no longer expressed as a single uninterrupted block but as a series of shorter segments separated by nonpolyester units.^96–98^

Backbone-embedded reactive groups therefore extend functionalization possibilities to synthesis routes with poor end-group control but inherently trade architectural simplicity and polyester sequence continuity for higher functional-group density and broader network topologies. section 3.1 exploits these different handle locations, terminal versus backbone, to install specific photoreactive motifs.

#### 2.2.3 Molecular-Weight Control and Chain-Length Distributions

The final structural feature inherited directly from the polyester synthesis is the chain-length distribution. For light-based 3D printing, polyesters are almost always used as low-molar mass oligomers rather than high-molar mass thermoplastics.^73,81–85^ In this regime, typically a few thousand g mol^–1^ for linear macromers and the corresponding per-arm values for star architectures, the chain-length distribution determines handle spacing, resin viscosity, miscibility, and ultimately the predictability of network formation.^92,100,102^

Critically, the importance of molar mass control is dependent on how photoreactive groups are introduced.

- For end-functionalized precursors, the molar mass directly sets the molar mass between cross-links (*M*_*c*_).^90,91^ Narrow dispersity therefore translates into uniform strand lengths, predictable network density, and reproducible mechanical behavior. Any dispersity in chain length produces a corresponding distribution in *M*_c_, a fundamental limitation of all synthetic polymers. Only sequence-defined polyesters, which contain a single, perfectly defined chain length and sequence, could in principle eliminate this distribution entirely and yield a uniquely well-defined network when combined with quantitative end-functionalization.^104^
- For backbone-functionalized precursors, by contrast, the density of reactive groups is primarily governed by comonomer incorporation rather than molar mass.^105,106^

The attainable molar mass and dispersity windows are dictated by the polymerization mechanism:

- **ROP** and **ROCOP**: living or pseudoliving behavior enables deterministic targeting of *M*_n_ via the monomer-to-initiator ratio. Narrow dispersities (<1.2) ensure tightly clustered chain lengths.^70–73,75^

- **Step-growth (polycondensation)**: Carothers’ relation (DP_n_ = 1/(1 – *p*)) dictates inherently broad distributions; lowering conversion to access low *M*_n_ does not narrow dispersity or impose uniform end-groups.^68^
- **rROP**: classical free-radical propagation with termination yields broad dispersities; coupling to controlled radical polymerization (CRP), such as reversible addition–fragmentation chain transfer (RAFT) polymerization can narrow dispersity.^18,80,81^

- **PHAs and enzymatic ROP**: molar mass is dictated by metabolic or enzymatic kinetics; dispersities are typically broad or multimodal unless fractionated postsynthesis.^86–89^

Across all mechanisms, the mechanistic origin of chain-length control directly governs printability. ROP and ROCOP provide the most robust foundations for end-functional oligomers used in light-based 3D printing, whereas step-growth routes, rROP, and biosynthetic pathways require compensatory design strategies, such as backbone-embedded reactive groups, mixed polymerization modes, or postfractionation, to approach comparable levels of control.^92,99,100^

## 3 From Polyester to Polyester Network by Light

The transition from a linear polyester to a cross-linked network marks a fundamental change in molecular architecture and macroscopic behavior. Linear polyesters derive their cohesion primarily from chain entanglements and secondary interactions, whereas a cross-linked polyester forms a permanent covalent network in which individual chains are linked into a single macromolecular entity.^5,7^ This network architecture suppresses viscous flow, imparts solvent resistance, enhances dimensional stability, and introduces elastic behavior that cannot be achieved with linear chains alone.

When photoreactive groups are incorporated into the polyester precursor, the formation of this network can be triggered with spatial and temporal precision using light.^11,12^ Irradiation, typically in the ultraviolet or visible range, initiates the chemistry that converts a fluid polyester-based formulation into a solid network. This light-addressable control over when and where cross-links form underpins the use of polyesters in light-based 3D printing and motivates a detailed examination of the chemical routes that enable such photoreactivity.^10–12^

This section therefore builds a conceptual bridge between polyester synthesis (section 2) and polyester 3D printing (section 4). First, we examine how photoreactive sites are introduced onto polyester precursors, either by postpolymerization functionalization or through copolymerization strategies (section 3.1).^6,10^ These reactive groups determine which photochemical pathways are accessible. Second, we describe the mechanistic families of photopolymerization, radical chain-growth, radical step-growth, and nonradical processes, and outline how each pathway governs network formation, topology, gelation behavior, oxygen sensitivity, and volumetric change (section 3.2).

Together, these subsections establish the molecular logic by which polyesters become photo-cross-linkable and provide the mechanistic framework required to understand how network formation constrains and enables light-based 3D printing of polyesters. The implications for printing performance, resin formulation, and final material properties are then developed in section 4.

### 3.1 Installation of Photoreactive Functionality

The introduction of photoreactive functionality is the chemical step that transforms linear or branched polyester chains into precursors capable of undergoing light-triggered network formation.^10,90,107,108^ Because most aliphatic polyesters are intrinsically inert toward photopolymerization, lacking both unsaturation and reactive side groups, photoreactivity must be deliberately installed either after or during polyester polymerization. As outlined in section 2.2, the polymerization mechanism determines whether this installation can proceed via uniform terminal groups, via backbone-embedded reactive groups, or via statistically distributed internal units; the present section now uses these structural possibilities to introduce specific photoreactive motifs.

Four structurally distinct strategies have emerged for installing photoreactive functionality on polyester precursors: (i) End-group modification introduces reactive units onto α,ω-hydroxyl termini using electrophiles, such as acyl derivatives, isocyanates, or epoxides (Figure 4a). (ii) Comonomer incorporation embeds photoreactive groups during polymerization through functionalized monomers, distributing reactive groups along the backbone according to the copolymerization statistics (Figure 4b). (iii) Initiator-mediated incorporation places a defined photoreactive unit at the α-terminus by using an initiator that bears the desired handle (Figure 4c). (iv) Backbone-directed derivatization introduces reactive motifs after polymerization at internal chain positions, independent of end-group fidelity (Figure 4d).

**Figure 4:**
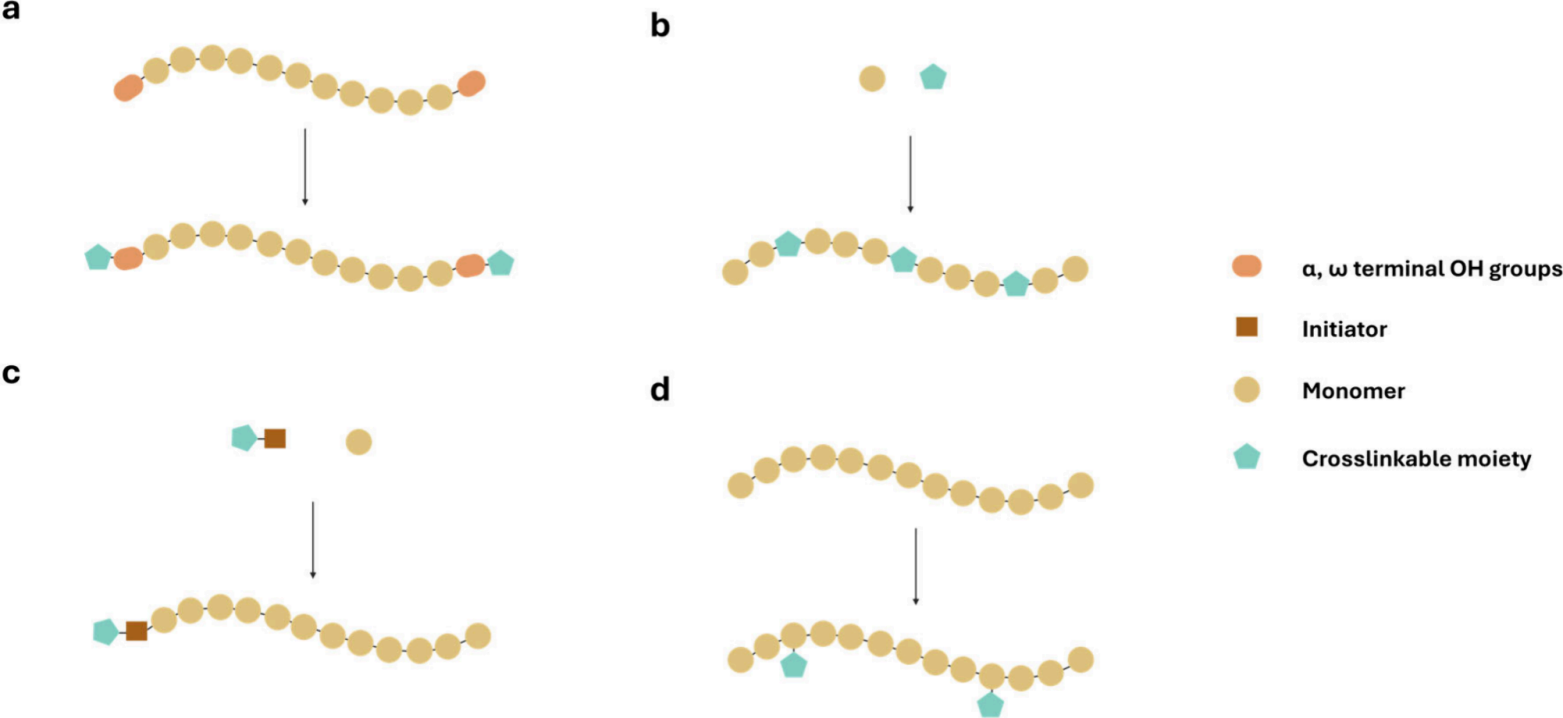
Schematic overview of the four principal strategies for installing photoreactive functionality onto polyester precursors. (a) End-group modification: reactive units are introduced at α,ω-hydroxyl termini using electrophiles such as acyl derivatives, isocyanates, or epoxides. (b) Comonomer incorporation: functional monomers introduce pendant photoreactive groups along the backbone according to copolymerization statistics. (c) Initiator-mediated incorporation: a photoreactive initiator embeds a single defined unit at the α-terminus during ring-opening polymerization. (d) Backbone-directed derivatization: postpolymerization reactions install reactive motifs at internal chain positions independent of end-group fidelity.

The following subsections examine these four strategies, emphasizing the underlying chemical transformations, scope, and practical considerations associated with each route. The consequences of these installation strategies for photopolymerization mechanisms, network topology, and printing behavior are discussed in sections 3.2 and 4.

#### 3.1.1 Functionalization of Terminal Hydroxyl Groups via Activated Electrophiles

Modification of terminal hydroxyl groups remains the most widely adopted strategy for introducing photoreactive functionality into polyesters. In practice, well-defined α,ω-diol architectures arise almost exclusively from ring-opening polymerization, where alcohol initiators and controlled transesterification permit precise placement of hydroxyl termini.^40,49^ Other polyester-forming routes can produce hydroxyl end-groups, but not with the structural definition or reliability required for consistent precursor design.^18,70,80^

The general principle underlying these transformations is nucleophilic substitution by the terminal alcohols on the suitably activated electrophiles. Depending on the electrophile class, this results in ester, urethane, or ether linkages that anchor acrylates, methacrylates, alkenes, thiols, or photocycloaddition motifs onto the polyester chain. The following subsections summarize these coupling strategies, emphasizing their mechanistic basis and practical consequences for resin preparation.

##### 3.1.1.1 Acyl Electrophiles

Acyl chlorides, anhydrides, and activated carboxylic acids react readily with the terminal hydroxyl groups of polyesters to generate ester-linked photoreactive functionalities. Reactions with acyl chlorides proceed via nucleophilic addition–elimination and typically reach high conversions under mild conditions due to the strong electrophilicity of the carbonyl carbon (Figure 5a,b). Their reactivity, however, demands strict control of moisture, and the neutralizing base used to capture liberated HCl often generates salts that require careful removal to avoid residual coloration in the final resin.^92–95,101,102,109–126^

**Figure 5:**
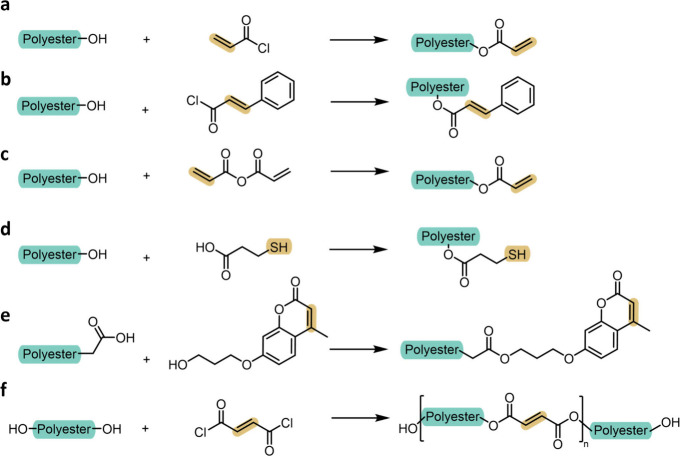
End-group modification of hydroxyl-terminated polyesters using acyl electrophiles. (a,b) Esterification via acyl chlorides, enabling efficient installation of (meth)­acrylates and other photoreactive groups. (c) Anhydride-mediated coupling, offering cleaner byproducts and improved resin clarity. (d) Acid-catalyzed functionalization of terminal hydroxyl to introduce terminal thiol functionalities. (e) Carbodiimide-activated esterification of carboxylic acid-bearing photoreactive units, including photocycloaddition motifs. (f) Chain-extension strategies using bifunctional acyl electrophiles (e.g., fumaryl chloride) to introduce conjugated linkers that subsequently undergo photochemical cross-linking.

Anhydrides constitute a less aggressive but synthetically convenient alternative (Figure 5c). They display lower intrinsic reactivity but offer cleaner byproducts and simplified purification, often yielding resins with higher optical clarity. Both acyl chloride- and anhydride-mediated reactions have been widely applied to introduce (meth)­acrylate groups,^100,103,127–132^ and cinnamate photocycloaddition units.^133^

In an alternative strategy, esterification of 3-mercaptopropionic acid catalyzed by *p*-toluene sulfonic acid enabled the installation of terminal thiol functionalities onto polyesters (Figure 5d).^134^ Furthermore, activated esterification protocols based on carbodiimides have been reported (Figure 5e). These reactions allow coupling of carboxylic acid–functionalized photocycloaddition motifs, such as coumarin, under mild conditions and without the need for highly reactive acyl derivatives.^135^

Finally, acyl electrophiles can also be used for chain-extension strategies (Figure 5f). For example, the reaction of fumaryl chloride with two terminal hydroxyl groups links polyester segments through a conjugated double bond that subsequently participates in photoinduced cross-linking.^136–138^ Such transformations illustrate the versatility of acyl electrophiles, which can install functionality either at chain termini or between polyester blocks.

##### 3.1.1.2 Isocyanates

Isocyanates provide a second electrophilic platform for hydroxyl functionalization, reacting with terminal alcohol groups to form urethane linkages under mild conditions.^99,139–146^ Both di- and monoisocyanates have been employed to introduce photoreactive moieties. Diisocyanates enable broad chemical flexibility but suffer from limited regioselectivity: either isocyanate group may react first, leading to partial chain extension or branching prior to attachment of the desired handle.^145^ While this complication reduces architectural precision, diisocyanate-mediated coupling has nonetheless been extensively used to graft acrylates, terminal alkenes, terminal alkynes, and photocycloaddition motifs onto polyesters (Figure 6a).^99,139–146^

**Figure 6:**
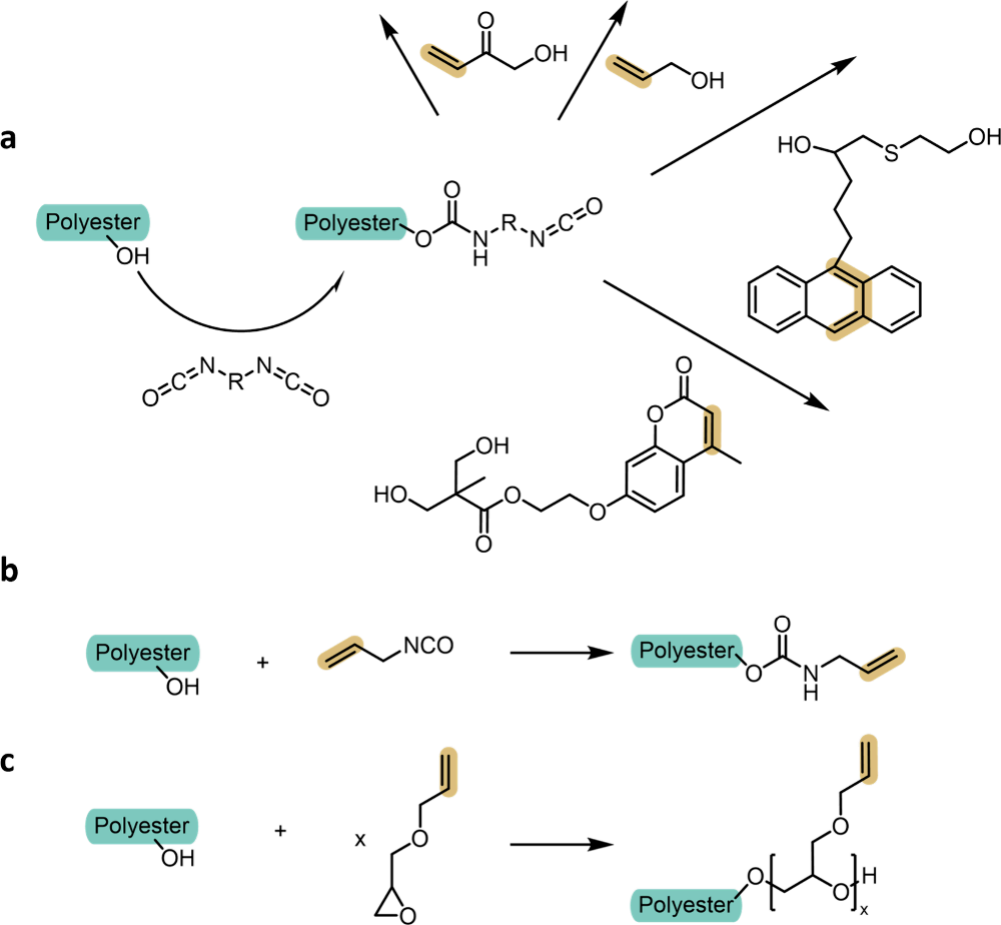
End-group functionalization of hydroxyl-terminated polyesters using activated electrophiles. (a) Urethane formation via diisocyanates, illustrating competitive chain extension prior to attachment of the photoreactive handle. (b) Monoisocyanate-mediated coupling, enabling selective installation of alkenes and other photoreactive groups with minimal molar mass perturbation. (c) Epoxide ring-opening by terminal alcohols to introduce ether-linked allyl functionalities under mild conditions.

To circumvent uncontrolled chain extension, monoisocyanates bearing a built-in photoreactive group offer a more selective alternative (Figure 6b).^91^ Their use enables the installation of terminal alkenes and other functionalities with improved control over the molar mass and degree of functionalization. The resulting urethane-linked photoreactive polyesters often exhibit high purity and well-defined functionality, provided moisture is rigorously excluded during synthesis.^90,91,145–148^

##### 3.1.1.3 Epoxides

Activated epoxides undergo nucleophilic ring opening by the terminal hydroxyl groups of polyesters, generating ether linkages and simultaneously introducing photoreactive functionality.^149,150^ This approach has been used to graft allyl groups onto hydroxyl-terminated chains by reacting with allyl glycidyl ether; the extent of functionalization can be tuned by adjusting reaction stoichiometry (Figure 6c).^9,96,151^ Epoxide opening proceeds under relatively mild conditions and avoids some of the color and purification issues associated with acyl chloride and isocyanate chemistry.

Together, these electrophile-driven end-group modification routes constitute the most direct and versatile approach for installing photoreactive sites on polyester chains. However, their reliance on well-defined hydroxyl termini limits their applicability to polymerizations that preserve end-group fidelity. The next section therefore examines strategies that incorporate photoreactive units directly during polymer formation, enabling functionalization independently of postsynthetic end-group modification.

#### 3.1.2 Incorporation of Photoreactive Moieties during Polyester Formation

Reactive groups may also be introduced directly during polyester synthesis, either by using functionalized comonomers or by initiating polymerization with a photoreactive alcohol.^9,85,152^ These approaches bypass reliance on terminal hydroxyl groups and enable access to architectures in which photoreactive units appear along the backbone or as pendant substituents, motifs that cannot be obtained through end-group modification alone. Because the spatial placement of these reactive groups follows the statistics of the polymerization mechanism, the resulting distribution is regular only in specific cases (such as perfectly alternating polymerizations) and more commonly statistical.^152–154^ The following subsections distinguish between incorporation through functional comonomers, which embed reactive groups throughout the chain, and incorporation through photoreactive initiators, which place a well-defined handle at the α-chain end.

##### 3.1.2.1 Incorporation via Functionalized Comonomers

Functionalized comonomers provide a direct means of embedding photoreactive groups into polyester chains. Here, the handle is built into the monomer itself and is introduced during chain growth, yielding backbones or pendant architectures that can later undergo light-induced cross-linking. Cyclic esters bearing activated double bonds, such as α-methylene-γ-butyrolactone, introduce pendant alkene groups suitable for radical curing (Figure 7a).^105^ Likewise, incorporation of glycidyl methacrylate through copolymerization with lactones yields polyesters containing pendant methacrylates that can participate in chain-growth photopolymerization (Figure 7b).^106^

**Figure 7:**
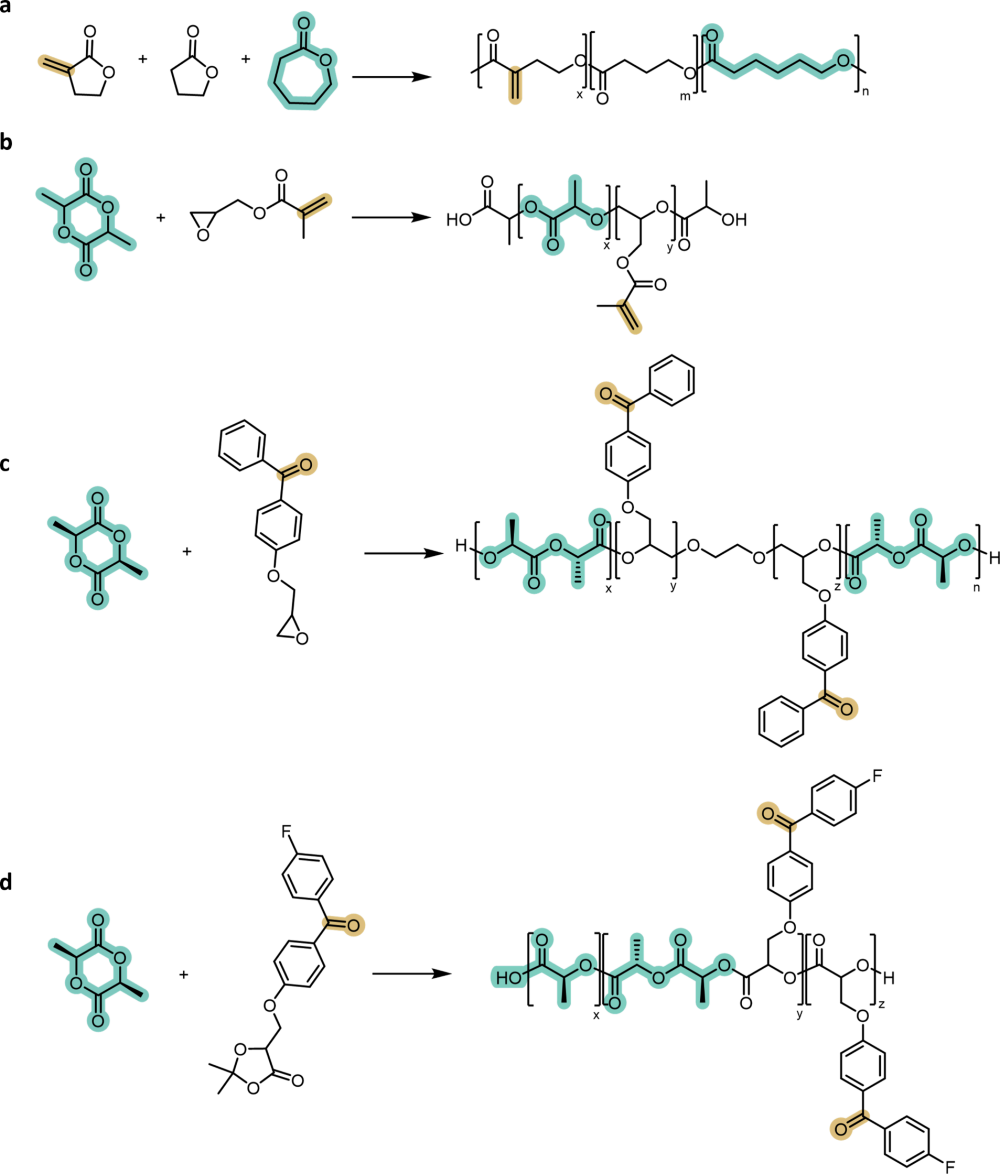
Integration of photoreactive groups into polyester backbones via functionalized comonomers. (a) Cyclic esters bearing activated double bonds (e.g., α-methylene-γ-butyrolactone) introduce pendant alkene groups for radical photopolymerization. (b) Copolymerization of glycidyl methacrylate with lactones yields polyesters carrying pendant methacrylate units. (c) Benzophenone-functionalized epoxides provide polyesters with repeat-unit-level photoinitiating motifs. (d) Benzophenone-substituted cyclic carbonates and related monomers introduce aromatic photo-cross-linking units while preserving aliphatic polyester degradability.

More complex photoactive motifs can also be introduced through appropriately substituted epoxides or carbonates. Benzophenone-functionalized monomers, for example, generate polyesters in which each repeat unit contains a benzophenone capable of initiating radical coupling upon irradiation (Figure 7c).^96,97^ Related strategies include benzophenone-substituted cyclic carbonates and dioxolanones, which provide modular access to polyesters carrying photoreactive aromatic units while retaining the degradability of the aliphatic backbone (Figure 7d).

Because the reactive groups are incorporated during chain growth, monomer compatibility with the polymerization mechanism and stability of the photoreactive group under reaction conditions remain the primary design constraints.^98^

##### 3.1.2.2 Incorporation via Functionalized Initiators

Photoreactive groups can also be incorporated into polyester chains through the use of functionalized initiators.^155–159^ Here, the initiator of the polymerization already bears the desired photoreactive handle, which becomes covalently embedded at the α-chain end during polymer growth. Because the initiator dictates the exact number and position of these groups, this route provides high-fidelity end-functionalization without relying on postpolymerization modification.

The principle is straightforward: a multifunctional alcohol carrying an acrylate, methacrylate, alkene, or aromatic photo-cross-linking unit initiates ring-opening polymerization, yielding polyesters that inherently terminate in a photoreactive group.^159^ For example, diols bearing acrylate functionalities have been employed to initiate the polymerization of lactide, generating polyester chains that carry a photoreactive segment derived directly from the initiator (Figure 8a).^160^ By incorporation of a rigid or aromatic initiator, additional structural features, such as increased backbone stiffness or enhanced absorption, can be introduced simultaneously with the reactive functionality.

**Figure 8:**
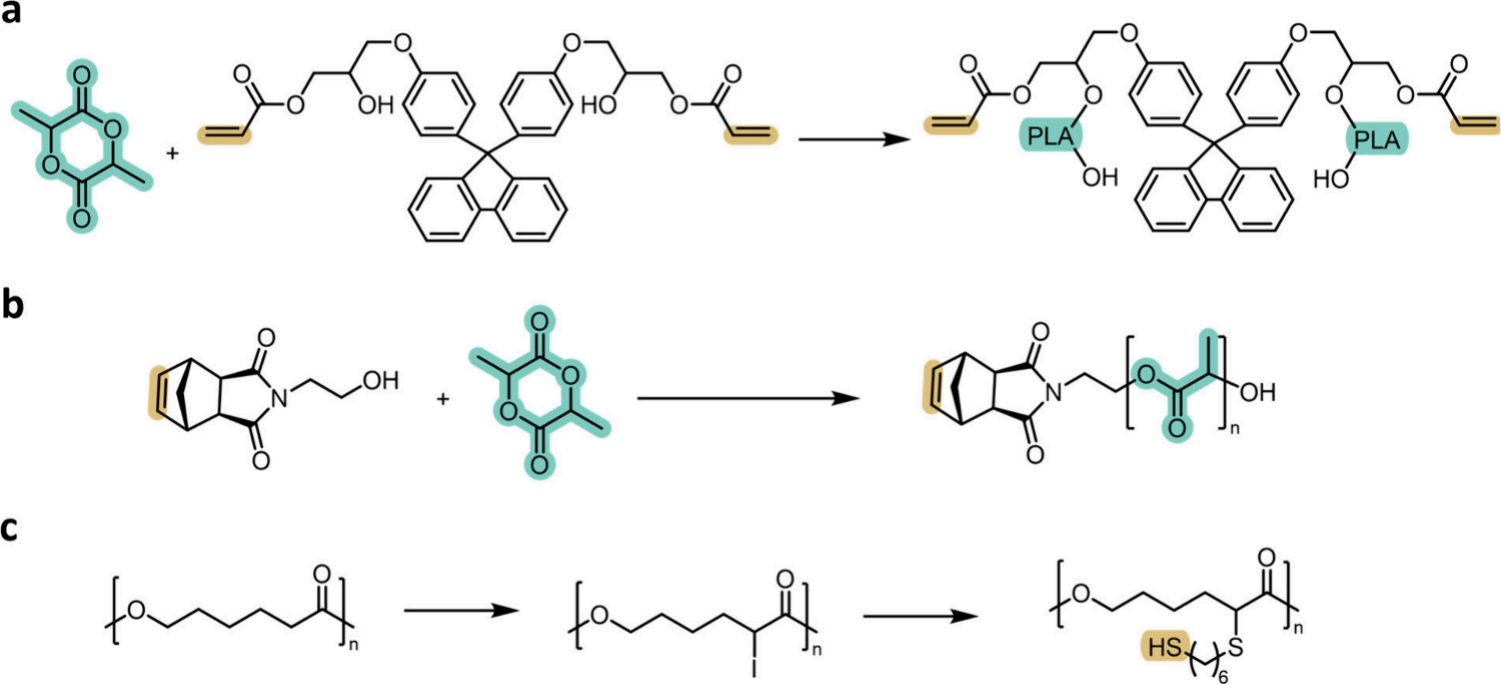
Incorporation of photoreactive functionality via initiator design and backbone halogenation. (a) Use of photoreactive alcohols as ROP initiators to embed acrylate units at the α-chain end of polyesters. (b) Photoreactive initiators enabling the synthesis of bottlebrush polyesters. (c) Backbone halogenation through lithiation/iodination, followed by nucleophilic substitution (e.g., difunctional thiols) to introduce internal thiol units for step-growth photopolymerization.

Initiator-mediated incorporation has also enabled the synthesis of more complex architectures. Photoreactive initiators have been used to generate bottlebrush polyesters. The bottlebrush polymers were obtained through polymerization of the α-chain end norbornene-functionalities following the polyester synthesis (Figure 8b).^161,162^ Because each polymer chain originates from a defined initiator, these strategies provide excellent control over the number and placement of photoreactive sites per macromolecule, ensuring uniformity across the polymer population. However, this precision comes with intrinsic limitations: the maximum number of reactive groups is fixed by the initiator structure itself, restricting the attainable functional-group density, and the synthesis of such initiators often requires multistep preparation, purification, and stability control. As a result, initiator-mediated routes offer unparalleled positional fidelity but are less flexible or scalable than end-group functionalization strategies that modify preformed polyesters.^10,108^

Although the library of photoreactive initiators is narrower than that of functional comonomers, initiator-mediated incorporation offers two clear advantages: (i) high precision in end-group identity due to the deterministic nature of initiation in controlled ROP, and (ii) the ability to introduce photoreactive groups without relying on the presence, fidelity, or reactivity of terminal hydroxyls—an important benefit for polyesters whose end-groups are statistically distributed or difficult to address through postpolymerization modification.

#### 3.1.3 Functionalization Strategies Independent of End-Group Fidelity

While most functionalization strategies rely on well-defined terminal hydroxyl groups or on incorporating reactive units during polymerization, certain transformations introduce photoreactive moieties directly onto the polyester backbone after chain formation. These methods are synthetically less common but offer a valuable alternative when end-group fidelity is compromised.^163,164^ Their defining feature is that the reactive handle is installed at internal chain positions rather than at chain termini, enabling access to architectures that cannot be obtained through electrophile-mediated end-group modification.

One example exploits the selective activation of backbone methylene units. In this strategy, the polyester is first lithiated to generate a backbone-bound organolithium species, which is subsequently quenched with a halogenating agent to yield an iodinated polyester (Figure 8c).^165^ The carbon–iodine bond then serves as a versatile handle for further nucleophilic substitution. Reaction with a difunctional thiol introduces terminal thiol groups along the backbone, producing thiol-functionalized polyesters capable of undergoing step-growth photopolymerization.^165^ Because the functional group is installed at internal positions, this approach remains applicable even when the original polymerization does not provide reliable control over the chain end identity.

Although reports of such backbone-level transformations are limited, they demonstrate that photoreactive motifs do not need to be restricted to chain ends or installed solely during polymerization. At the same time, these reactions inherently offer less control than end-group modification or comonomer incorporation: the extent and spatial distribution of substitution are difficult to regulate, and each modification interrupts the native polyester sequence.

With the installation strategies defined, section 3.2 examines how the resulting photopolymerization mechanisms construct polyester networks and how these networks constrain the light-based 3D printing behavior developed in section 4.

### 3.2 Photopolymerization Chemistry and Network Consequences

The installation of photoreactive functionality on polyester precursors defines which chemical pathways become available upon irradiation, but it is the underlying photopolymerization mechanism that determines how these precursors assemble into a cross-linked network. Each photochemical family, radical chain-growth, radical step-growth, and nonradical pathways, creates networks through fundamentally different modes of bond formation, producing characteristic patterns of gelation, cross-link density, homogeneity, oxygen sensitivity, and volumetric change.^166–169^ These mechanistic signatures arise directly from how reactive intermediates are generated, how they propagate, and how they terminate, and they shape the intrinsic architecture of the resulting polyester network before factors such as formulation, viscosity, or printing modality intervene.^10,103,108,169,170^

This section therefore examines photopolymerization at the level of the chemical mechanism. The aim is not to discuss printing performance, that analysis is developed in section 4, but rather to establish how each mechanistic family constructs a network and why their architectures differ. By doing so, the discussion provides the conceptual framework needed to interpret how the photochemistry introduced in section 3.1 determines the behavior of cross-linked polyesters once exposed to light. The discussion in this section proceeds in two layers. The first considers the photopolymerization families themselves, radical chain-growth, radical step-growth, and nonradical pathways, and how light activation generates reactive intermediates and new covalent bonds. The second translates these mechanisms into quantities that are decisive for light-based 3D printing: gelation behavior, cure kinetics, oxygen inhibition, and volumetric shrinkage.

#### 3.2.1 Mechanistic Signatures of Photopolymerization Families

##### 3.2.1.1 Chain-Growth Network Formation

Radical chain-growth polymerization of (meth)­acrylates represents the archetypal photopolymerization mechanism and remains the most widely applied route for constructing polyester-based networks.

In the initiation step, light-induced cleavage of a photoinitiator yields primary radicals that add to the C=C bond of a (meth)­acrylate moiety, generating the first propagating species. Propagation involves successive radical additions to pendant vinyl groups, extending the chain (Figure 9). Termination occurs either by radical–radical combination, forming a single covalent bond, or by disproportionation, producing a pair of unsaturated and saturated chain ends. When multifunctional monomers or macromers are used, propagation rapidly links multiple chains, giving rise to a cross-linked network.^92,166^

**Figure 9:**
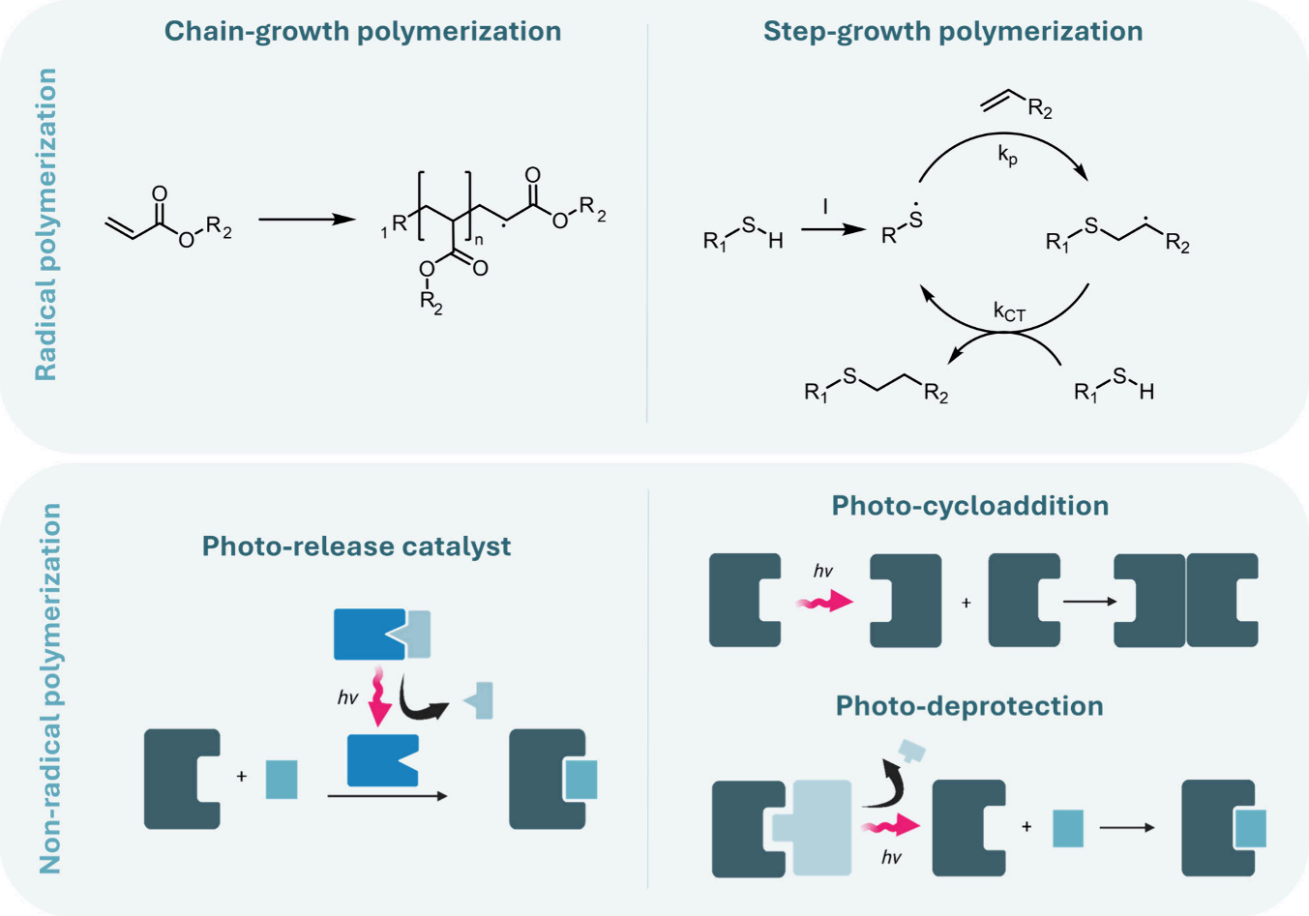
Photopolymerization mechanisms relevant to polyester network formation. Schematic comparison of the three mechanistic families that generate cross-linked polyester networks upon irradiation. (top left) Radical chain-growth polymerization of (meth)­acrylates. (top right) Radical step-growth photopolymerization, exemplified by the thiol–ene reaction. (bottom left) Photolysis-triggered catalysis. (bottom right) Excited-state photocycloadditions (top) and photouncaging of a nucleophile (bottom).

##### 3.2.1.2 Step-Growth Network Formation

Radical step-growth photopolymerizations construct networks through architectural logic different from that of radical chain-growth systems, even though both share mostly the same basic photochemical steps (Figure 9). Instead of a single propagating chain end that sequentially incorporates monomer units, step-growth systems form bonds through discrete, bimolecular reactions between complementary functional groups. The thiol–ene reaction is the prototypical example and illustrates the mechanistic logic that distinguishes step-growth pathways from chain-growth mechanisms.^167–169^

Thiol–ene photopolymerization proceeds through the same elementary radical steps, initiation, propagation, and termination, but with a crucial mechanistic distinction: each radical addition to an alkene is immediately followed by a chain-transfer event in which the carbon-centered radical abstracts a hydrogen atom from a thiol. This regenerates a thiyl radical and terminates growth of the transient carbon-centered species. As a result, the radical center is continuously passed between chains rather than residing on a growing chain end. Because no persistent propagating chain is formed, all functional groups participate more uniformly, and bond formation is distributed statistically throughout the system.^168^

The relative rates of propagation and chain-transfer define whether a system behaves as a true step-growth polymerization or as a mixed-mode mechanism. When the propagation and chain-transfer rate constants are comparable, as in thiol–norbornene formulations, the reaction follows ideal step-growth behavior. When propagation significantly outpaces chain transfer, as in thiol–acrylate mixtures, chain-growth character emerges, and the resulting network reflects a hybrid of the two mechanisms.^171,172^

##### 3.2.1.3 Nonradical Network Formation

Nonradical photochemical pathways encompass several mechanistically distinct routes to network formation, unified by the absence of a propagating radical but differing profoundly in how light participates in bond formation. Three nonradical polymerization strategies exist to date via which cross-linked networks can be created upon irradiation with light: excited-state photocycloadditions, photochemical release of nucleophiles, and photochemical release of catalysts (Figure 9). These mechanisms occupy a small but conceptually important space in polyester photochemistry: they enable network formation without introducing carbon–carbon linkages, allow the incorporation of reversible or degradable motifs, facilitate the design of chemically orthogonal systems, and create opportunities for multiwavelength activation. Their relevance to light-based 3D printing is therefore not in replacing radical polymerization, whose speed and efficiency remain unmatched, but in defining an orthogonal set of chemical principles that could complement or extend the design space of polyester-derived photoresins.^12,173,174^

Photocycloadditions constitute the oldest class of nonradical photochemical cross-linking reactions. Here, light directly excites a chromophore, commonly cinnamate, coumarin, or anthracene derivatives, into a reactive excited state that undergoes a bimolecular [2 + 2] or [4 + 4] cycloaddition.^133,135,175–178^ Each productive event therefore requires a photon, and each photon yields at most one covalent cross-link. Because no propagating intermediate is formed, network assembly proceeds through discrete photochemical encounters between excited chromophores and strand formation reflects the spatial probability of these encounters rather than kinetic chain growth.

A mechanistically distinct pathway arises when light does not participate directly in bond formation but instead unblocks a nucleophile.^179^ In photocaged-nucleophile systems, photolysis cleaves a protecting group to release one equivalent of the nucleophile, which then reacts once with an electrophile. No catalytic species is formed, no propagation occurs, and each bond again requires a separate photolysis event. Although these reactions do not rely on electronically excited intermediates after photolysis, their photon-limited stoichiometry aligns them more closely with photocycloadditions than with photopolymerizations that involve sustained propagation.

The third family, latent-catalyst photopolymerizations, operates through a different mechanistic logic. Photoacid generators (PAGs) and photobase generators (PBGs) undergo photolytic cleavage to release an acid or a base that subsequently drives a purely thermal polymerization.^179–184^ In these systems, light serves only to generate the catalytically active species; all ensuing bond-forming steps proceed without further photon involvement. The resulting polymerization mechanism, therefore, depends entirely on the ground-state reactivity of the unmasked catalyst and the functionality of the monomers. PAGs typically initiate cationic chain-growth reactions, most prominently the ring-opening polymerization of strained cyclic ethers, where propagation proceeds through oxonium intermediates and yields long kinetic segments. A pivotal demonstration of this approach was provided by Kojic et al. in 2023, who reported the first light-based fabrication of poly­(ether-ester) architectures using a fully nonradical mechanism.^185^ PBGs and photocaged bases, by contrast, often initiate step-growth processes such as thiol–isocyanate, thiol–epoxy, or base-catalyzed Michael additions, where network growth arises from distributed bimolecular reactions rather than from a propagating chain. In both cases, a single photolysis event generates a catalyst that can form many bonds, giving these pathways a photon economy fundamentally different from those of both photocycloadditions and photocaged-nucleophile systems.

Together, these mechanisms define three photochemical modes, excited-state cross-linking, stoichiometric photolysis, and photolysis-triggered catalysis, each imposing characteristic patterns of bond formation and photon efficiency. These mechanistic distinctions establish the intrinsic connectivity and homogeneity of the emerging networks and provide the foundation for analyzing their gelation behavior, oxygen response, and volumetric evolution in section 3.2.2.

#### 3.2.2 Network Considerations for Light-Based 3D Printing of Polyesters

##### 3.2.2.1 Network Emergence: Gelation, Topology, Homogeneity, and Cross-link Density

The emergence of a covalent network from initially mobile polyester macromers is governed most directly by the photopolymerization mechanism. Whether a precursor cures via radical chain-growth, radical step-growth, or a nonradical pathway, the way reactive intermediates are generated and consumed dictates how quickly a sample-spanning network forms, how homogeneous that network becomes, and how cross-links are distributed in space.

In multifunctional chain-growth polymerizations, gelation occurs at low conversion, because connectivity develops along the trajectories of the actively propagating chains rather than through uniform consumption of functional groups across the entire precursor population. As these kinetic chains continue to add nearby vinyl groups, local regions of high connectivity emerge early in the reaction, enabling the formation of a percolated network at only ∼10–20% overall double-bond conversion.^186^ This mode of growth yields networks in which strand lengths and junction connectivities reflect the stochastic propagation history of individual chains, leading to distributions that are broader than those obtained from mechanisms that rely on uniform, pairwise functional-group consumption.

Radical step-growth polymerizations, exemplified by thiol–ene systems, construct networks through an entirely different architectural logic.^166,187–190^ Here, each thiyl radical adds to an alkene, and the resulting carbon-centered radical immediately undergoes hydrogen abstraction from a thiol. This alternating addition–transfer sequence continuously shifts the radical center between molecules and prevents the formation of any persistent growing chain end. Bond formation therefore arises from many statistically distributed bimolecular reactions between complementary groups.

Connectivity in step-growth systems increases gradually, and gelation occurs only when a sufficiently large fraction of functional groups has reacted, at substantially higher double-bond conversion than in chain-growth systems (Figure 10).^186^ Because each functional group experiences a similar probability of reaction, the resulting networks exhibit narrower strand-length distributions and greater topological homogeneity than their chain-growth counterparts.

**Figure 10:**
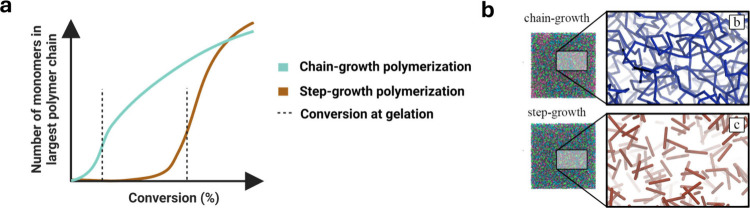
(a) Growth of the largest polymer unit as a function of double bond conversion for step- and chain-growth polymerizations. Vertical dashed lines indicate the gelation point for each system, emphasizing the lower gelation threshold of chain-growth mechanisms. (b) Schematic representation of the resulting network topologies. Chain-growth polymerization yields a heterogeneous network with early gelation, while step-growth polymerization forms a more homogeneous network structure. Adapted with permission from ref 186. Copyright 2023 American Chemical Society.^186^

For such statistically uniform networks, classical rubber elasticity can be invoked to relate the elastic modulus to the molar mass between cross-links,

1 $$G^{\prime }=\frac{\rho RT}{M_{c}}\left(1-\frac{2}{f}\right)\left(1-\gamma \right)$$

where *M*_*c*_ is the average molar mass between cross-links, *f* the junction functionality, and γ the defect fraction. Chain-growth networks, by contrast, deviate strongly from this idealized behavior, as no single characteristic *M*_*c*_ describes their broad distribution of strand lengths.^191–194^

A direct mechanistic consequence emerges when the number of network-connecting bonds formed per reactive alkene. In radical chain-growth polymerizations, each vinyl group incorporated during propagation generates two σ-bonds that connect the growing chain to the surrounding strands. In thiol–ene step-growth reactions, each alkene forms one network-connecting thioether bond, while the companion bond-forming step yields a terminal C–H bond that does not contribute to connectivity.^144,168^ Thus, under equal initial double-bond concentrations, chain-growth systems can, in principle, generate approximately twice as many network-connecting linkages as step-growth systems. This stoichiometric asymmetry underlies the typically higher modulus and higher attainable cross-link density of acrylate networks and reinforces that mechanical comparisons between acrylate and thiol–ene systems must account for these intrinsic differences in bond-formation stoichiometry.

Nonradical pathways build connectivity according to the photon economy and ground-state chemistry described in section 3.2.1. Photocycloadditions and photocaged-nucleophile systems increase connectivity stoichiometrically, with each photochemical event contributing at most one new cross-link; gelation is therefore photon-limited and typically requires high chromophore or functional-group densities.^135,176,180,195^ By contrast, latent-catalyst systems (photoacid and photobase generators) decouple photon input from bond count: a single photolysis event can initiate many thermal bond-forming steps.^180,196,197^ Architecturally, such catalyst-triggered networks can approach the homogeneity of step-growth systems, but their temporal evolution is dictated by the ground-state kinetics of the unmasked catalyst rather than radical propagation.

These mechanistic architectures define the structural envelope within which oxygen inhibition, volumetric change, and cure kinetics operate. The following sections (section 3.2.2.2–3.2.2.4) analyze how each mechanistic family responds to those additional constraints once irradiation begins.

##### 3.2.2.2 Polymerization Kinetics

The preceding discussion addressed how different photopolymerization mechanisms construct networks; the next question is how fast these mechanistic patterns develop once light is applied. The rate at which connectivity emerges depends on the intrinsic photokinetics of the functional groups introduced in section 3.1 and determines how rapidly reactive species are generated and how steeply conversion evolves. Because photopolymerization kinetics are a field of study in their own right, particularly for (meth)­acrylates, thiol–ene reactions, and nonradical photoprocesses, this discussion does not attempt to be comprehensive but instead highlights those kinetic features that are most relevant for polyester-based photoresins.

Polymerization kinetics in polyester-based photoresins can be decomposed into two interconnected processes: the photophysical generation of reactive species and the intrinsic chemical reactivity of the functional groups installed on the polyester precursor. The first kinetic layer is defined by how efficiently irradiation generates the species responsible for bond formation. This depends on how much light reaches the chromophore, how strongly the chromophore absorbs at the irradiation wavelength, and how effectively the absorbed photon is converted into a radical (or other reactive intermediate).^10,107^ These three parameters determine the photon-to-reactivity efficiency and, therefore, how sensitively a resin responds to the dose distributions imposed by a printing workflow.

Once reactive species are formed, the second kinetic layer reflects the chemical reactivity of the specific functional group. For polyester-based systems this is dominated by acrylates, methacrylates, and thiol–ene reactions, which differ systematically in their propagation behavior.^94,168,171^ Acrylates generally react fastest as compared to methacrylates because their electron-poor, unhindered vinyl groups undergo efficient radical addition and support rapid propagation through stabilized secondary radicals. Methacrylates, by contrast, contain an α-methyl substituent that reduces radical addition rates both sterically and electronically; the tertiary radical formed after addition is more stabilized, which slows propagation.

Thiol–ene reactions follow a distinct kinetic logic, where every radical addition to an alkene is immediately followed by hydrogen abstraction from a thiol, which regenerates a thiyl radical and prevents the accumulation of any long-lived propagating species. Because of this alternating addition–transfer sequence, the overall rate depends on the structure of both partners. Electron-rich alkenes such as vinyl ethers and allyl ethers undergo rapid thiyl addition, and strained cyclic alkenes such as norbornenes react particularly quickly due to their highly activated double bonds.^169^ Electron-poor or conjugated alkenes react more slowly, because thiyl addition is less favorable. On the thiol side, primary alkanethiols abstract more readily than sterically hindered or aromatic thiols, and thiols with acidic S–H bonds accelerate the hydrogen-transfer step. These structure–reactivity relationships define the broad kinetic range observed experimentally. Several thiol–ene formulations reach conversions comparable to acrylates under practical exposure conditions, but their temporal profiles differ: step-growth thiol–ene networks typically exhibit more gradual rate changes and reduced autoacceleration, reflecting the chain-transfer-dominated mechanism and the absence of the pronounced Trommsdorff–Norrish effect characteristic of (meth)­acrylate chain-growth systems.^171,198,199^

Beyond these radical processes, nonradical photochemical pathways exhibit far more diverse kinetic regimes because the underlying mechanisms include cationic ring-opening, nucleophile-initiated additions, thiol–Michael and thiol–isocyanate reactions, and a range of excited-state photochemical transformations.^180,195,197^ Their rates depend strongly on the catalyst or nucleophile formed photochemically and on the thermal bond-forming steps that follow. Some ionic or nucleophilic systems can reach rates comparable to fast radical chemistries, but many proceed more slowly under printing-relevant conditions.^197^ A notable exception in the opposite direction is photocycloadditions, where low quantum yields and the requirement that each covalent bond-forming event consume a photon impose intrinsically slow, strictly photon-limited kinetics.^176^ This low photon efficiency, rather than the inherent chemistry of the cycloaddition, is the principal barrier to their use in rapid light-based printing.

Overall, the kinetic behavior relevant to polyester photopolymerization is therefore defined first by the efficiency with which light generates the active species and second by the chemical reactivity of the acrylate, methacrylate, or thiol–ene units (or, less commonly, the slower nonradical pathways) once these species are present. These two layers determine how quickly functional groups introduced in section 3.1 participate in the network formation mechanisms described in section 3.2 and set the time scale on which polyester resins respond to the spatial and temporal dose patterns imposed during printing.

##### 3.2.2.3 Oxygen Inhibition

Oxygen inhibition sits at the intersection of the polymerization mechanism and printing performance. Because molecular oxygen reacts with most carbon-centered radicals with diffusion-controlled rate constants (*k* ≈ 10^8^–10^9^ M^–1^ s^–1^, typically faster than propagation), its presence directly shapes cure thresholds, dose–conversion behavior, and spatial fidelity across the printing modalities discussed in section 4.1.

In classical (meth)­acrylate chain-growth polymerizations, oxygen reacts with both primary and propagating radicals to generate peroxyl species that do not propagate.^170^ Polymerization can proceed only after local oxygen has been depleted, giving rise to an induction period and a finite critical dose before any measurable conversion occurs. In SLA and DLP, this reduces cure depth and can weaken interlayer adhesion.^200,201^ The same chemistry is used constructively in CLIP: continuous oxygen permeation maintains a thin region in which radicals are quenched faster than they can propagate, preventing adhesion to the window and enabling continuous replenishment. Without strong oxygen inhibition, the dead zone cannot form and continuous printing collapses. Furthermore, as (meth)­acrylate polymerization starts only after local oxygen is consumed, inhibition creates a dose threshold that helps distinguish in-part from out-of-part regions during tomographic dose accumulation.^12,202,203^

Thiol–ene step-growth systems interact with oxygen through a different kinetic sequence.^170,172^ Oxygen intercepts carbon-centered intermediates with the same fast rate constants, but the resulting peroxyl radicals abstract hydrogen from thiols, regenerating thiyl radicals and converting oxygen into hydroperoxides. Because this reaction restores a reactive intermediate to the thiol–ene cycle, polymerization proceeds efficiently in air with minimal or no induction period. Thiol–ene curing therefore leads to consistent conversion even at low photon flux and retains interlayer adhesion without oxygen control. This oxygen tolerance is advantageous in most layerwise modalities but incompatible with CLIP, where the rapid consumption of oxygen prevents formation of the required inhibition layer. In volumetric printing, thiol–ene resins often require deliberate introduction of persistent radical inhibitors (e.g., (2,2,6,6-tetramethylpiperidin-1-yl)­oxyl, i.e., TEMPO) to re-establish a thresholded dose–conversion response.^12^

Photocycloaddition-based systems experience oxygen through an entirely different pathway. Here, oxygen quenches the triplet excited states of coumarin, cinnamate, or anthracene chromophores, shortening their lifetimes and reducing the quantum yield. No peroxyl inhibition pathway is involved, but the effect on the efficiency is substantial: long exposures or oxygen-controlled conditions are often required. As a result, these systems are highly sensitive to dissolved oxygen at the excitation step rather than at the propagation step.^170,176^

Across all mechanisms, the essential point is that oxygen inhibition is not a peripheral constraint but rather a mechanistic determinant of whether a given polyester photoresin can function under the optical, spatial, and kinetic requirements of a specific 3D printing modality. Its influence emerges not from the polymer itself but from the fate of the reactive intermediates that govern network formation and the printing consequences that follow directly from their elementary reaction pathways.

##### 3.2.2.4 Polymerization Shrinkage

Polymerization shrinkage is one of the most direct ways in which network chemistry manifests on the scale of the printed object. When a liquid resin is converted into a cross-linked solid, molecules that previously interacted only through van der Waals forces become locked together by covalent bonds. The average distance between segments decreases, the free volume is reduced, and the material densifies. For light-based 3D printing, shrinkage sets how closely a printed part can reproduce its CAD geometry, how much internal stress is frozen in during curing, and how strongly thin features or lattices warp or distort after printing.^107,171,172^ Because shrinkage is governed by how many covalent connections are created per unit volume, and how and when they form, it is intrinsically linked to the photopolymerization mechanism and to the way photoreactive groups are distributed along the polyester chains.^173,204^

In radical chain-growth systems based on (meth)­acrylates, shrinkage is typically the largest. The resins are rich in small, highly functional monomers and macromers; many double bonds are converted in a small volume, and each propagation event ties previously independent segments into a growing network. Gelation occurs early, so the material becomes mechanically rigid, while a substantial fraction of reactive groups is still unreacted. As curing continues, further covalent links are formed in the already solidifying matrix. The resulting contraction can no longer be accommodated by flow or local rearrangement and is instead stored as internal stress.^107,166,171^

Radical thiol–ene step-growth networks exhibit the same basic densification process, but the way this contraction manifests is different. Bond formation is distributed statistically throughout the volume and gelation is delayed to high overall conversion, so much of the shrinkage occurs while the material is still mobile enough to relax.^164,168,180^ In addition, each reactive alkene in a thiol–ene system contributes only one network-connecting thioether bond, whereas each acrylate incorporation in a chain-growth mechanism yields two σ-bonds that tie neighboring segments together. This lower intrinsic connectivity per double bond reduces the volumetric densification generated at a given functional-group conversion. The net shrinkage in thiol–ene systems can still be appreciable, but it is more homogeneous, evolves later in the cure, and is less tightly coupled to the moment of gelation. These combined features underlie the typically lower residual stress and improved dimensional stability of thiol–ene networks relative to acrylate analogues of comparable precursor molar mass.^107,173^

A second lever, which is independent of the mechanism, is where and how densely photoreactive groups are placed on the polyester. Low-molar mass, highly functional macromers place many reactive units in close proximity: curing then introduces a large number of covalent links per chain and per unit volume, maximizing shrinkage and often suppressing thermoplastic-like features such as crystallinity or ductility.^174,175^ Longer polyester segments between reactive sites, or architectures with fewer reactive groups per chain, reduce the number of cross-links formed within a given volume and allow the underlying polyester to retain more of its original behavior.^176,177^ In this sense, shrinkage is as much a function of the functionalization strategy and the chain-length window defined in section 2 as it is of the photochemical pathway itself.

A qualitatively different situation arises in ring-opening photoreactions, where bond formation is coupled to the opening of a strained or cyclic unit. When an epoxide, cyclic carbonate, or related motif opens during photoinitiated curing, part of the volumetric loss associated with the creation of covalent bonds can be offset by the release of the ring strain.^185–187^ The net shrinkage then reflects a balance between densification through cross-linking and expansion through ring-opening. This balance can be tuned by monomer design and is one of the reasons why nonradical or cationically triggered epoxide-based systems are attractive for applications where dimensional accuracy is critical.^185,187^ For polyester-based networks, the same design logic could be exploited by integrating ring-opening motifs into the photo-cross-linking chemistry, allowing shrinkage reduction without abandoning fully degradable backbones.

Across all of these cases, the central point is that shrinkage is not an isolated materials parameter; it is the outcome of three coupled design decisions: the photopolymerization mechanism, the density and placement of photoreactive motifs on the polyester, and the exposure scheme imposed by the printing modality (section 4.1). Controlling shrinkage therefore means designing how and when covalent connections are introduced, not merely compensating for dimensional change after the fact.^204^

These mechanistic and topological boundaries define the envelope within which polyester resins must operate during printing, and they form the structural basis for the modality-specific constraints analyzed in section 4.

## 4 From Network Formation to Light-Based 3D Printing of Polyesters

The transition from covalent network formation to light-based 3D printing introduces a new set of constraints that arise not from polymer chemistry alone but from the way light is delivered, accumulated, and translated into spatially selective cure. Whereas the preceding sections established how polyester precursors are synthesized and converted into covalent networks, the performance of these networks in an actual printer depends critically on the hardware-specific exposure geometry, the kinetics imposed by the illumination scheme, and the physical requirements placed on the resin during the brief interval between photon absorption and gelation. Light-based 3D printing encompasses a spectrum of modalities that differ fundamentally in how they generate three-dimensional dose patterns, from layered or patterned illumination in single-photon systems to focal-volume confinement in multiphoton methods to spatially integrated dose fields in volumetric approaches. These differences define distinct operational windows for viscosity, optical clarity, cure depth, and polymerization speed, all of which determine whether a given polyester formulation can be processed into high-fidelity objects. The following subsection provides a concise overview of the major printing modalities, outlining the exposure principles that later govern both the historical evolution of polyester-based printing (section 4.2) and the resin-level design constraints discussed in section 4.3.

### 4.1 Light-Based 3D Printing: A Short Discussion of the General Modalities

Light-based 3D printing encompasses a diverse set of technologies that implement light-induced polymerization through distinct hardware configurations and exposure strategies. While all rely on the same fundamental photochemical processes, the manner in which light is delivered, whether layerwise through patterned illumination, pointwise through tightly focused beams, or spatially integrated through volumetric dose, gives rise to characteristic differences in attainable resolution, printing speed, and geometric complexity. These modalities span spatial resolutions from the nanometer scale in two-photon polymerization to the micrometer scale in tomographic volumetric printing, with corresponding variation in manufacturing time ranging from precision serial writing to ultrafast volumetric fabrication.^10–12,107,204^

The following discussion briefly outlines the operating principles and exposure characteristics of the most widely used techniques, stereolithography (SLA), digital light processing (DLP), continuous liquid interface production (CLIP), two-photon polymerization (2PP), tomographic volumetric printing (VP), and light-sheet-based approaches, supported by a schematic overview in Figure 11 and detailed reviews elsewhere.^10–12,107,204^

**Figure 11:**
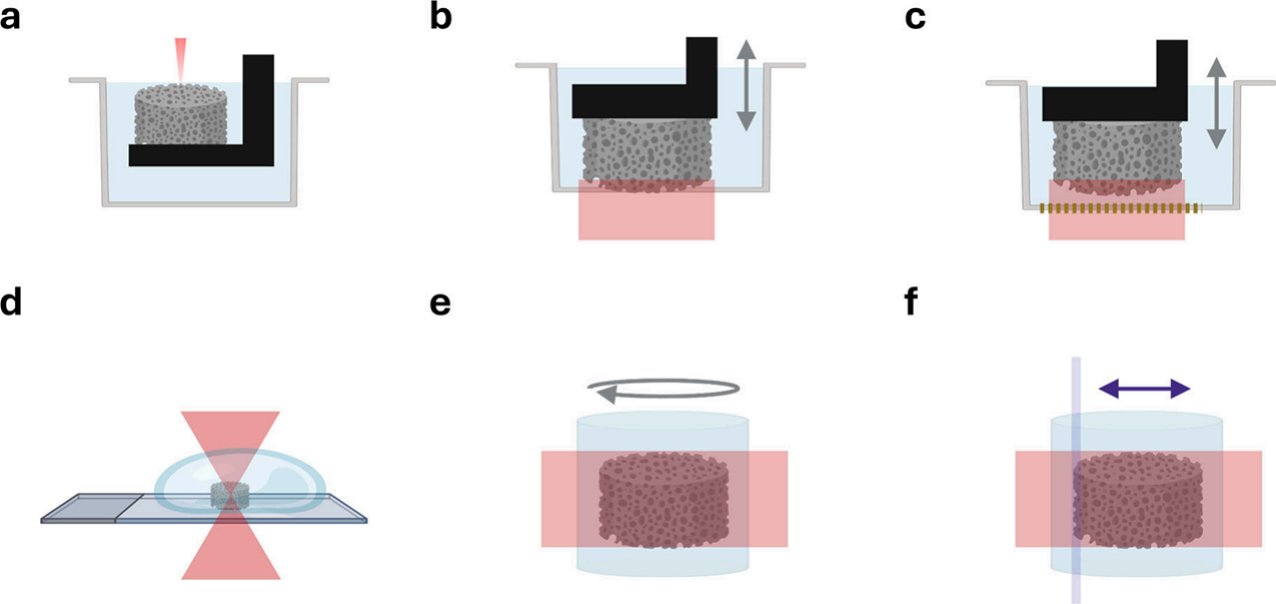
Overview of currently available light-based 3D printing technologies. (a) Stereolithography (SLA), (b) digital light processing (DLP), (c) continuous liquid interface production (CLIP), (d) two-photon polymerization (2PP), (e) tomographic volumetric 3D printing (VP) (also referred to as computed axial lithography or CAL), and (f) light-sheet volumetric 3D printing (also referred to as xolography).

#### 4.1.1 Single-Photon 3D Printing

Single-photon light-based 3D printing comprises the most widely implemented additive manufacturing modalities and remains the technological foundation from which many subsequent methods have evolved. In all variants, polymerization is triggered through the absorption of a single photon by a photoinitiator and light is delivered either as a scanned point or as a projected pattern. Although these techniques differ significantly in how light is spatially applied, they all accumulate dose layerwise, which defines both the attainable resolution and printing speed.

Stereolithography (SLA) represents the earliest implementation of this principle. First demonstrated in the early 1980s by Kodama and later by Hull,^205,206^ SLA constructs objects through point-by-point writing, in which a focused laser beam is rastered across the resin surface via a scanning mirror (Figure 11a). The vertical resolution is governed by the layer thickness, while the lateral resolution depends on the beam waist of the focused laser, rendering SLA inherently anisotropic in its spatial precision. Decades of improvements, including microstereolithography (μSLA) introduced in the 1990s, have steadily pushed achievable feature sizes into the low-micrometer regime.^207,208^ SLA thus offers high precision but is limited in speed by the serial nature of the exposure.

Digital light processing (DLP), introduced by Bertsch and co-workers in 1996,^207^ addressed this limitation by moving from pointwise to patternwise exposure. Early implementations used liquid-crystal displays (LCDs) as dynamic masks, but the transition to digital micromirror devices (DMDs) provided both higher resolution and improved optical throughput (Figure 11b). In DLP, an entire layer is polymerized simultaneously and the printing speed scales primarily with the number of layers. Lateral resolution is defined by the pixel pitch of the micromirror array and is typically comparable to that of SLA, while overall throughput is significantly higher, because of the parallelized exposure.

Continuous liquid interface production (CLIP), introduced by Tumbleston et al. in 2015,^209^ further accelerated single-photon printing by eliminating the mechanical separation step between layers. CLIP relies on an oxygen-permeable window at the bottom of the resin vat, which sustains a thin “dead zone” where polymerization is inhibited through radical quenching. This oxygen-rich layer prevents adhesion between the growing part and the window, allowing continuous replenishment of uncured resin beneath the printed object and enabling uninterrupted upward motion during exposure (Figure 11c).^200,201^ As a result, CLIP achieves printing speeds markedly higher than conventional DLP while maintaining comparable resolution, making it a relevant approach for applications requiring the rapid production of large or monolithic components.

#### 4.1.2 Two- and Multiphoton 3D Printing

Two- and multiphoton 3D printing exploits nonlinear optical absorption processes to achieve spatial confinement of polymerization beyond the diffraction limit of single-photon techniques. Among these, two-photon polymerization (2PP) is the most widely implemented. Developed conceptually in the 1970s and experimentally realized for microfabrication in the 1990s, 2PP relies on the near-simultaneous absorption of two photons by a photoinitiator molecule, typically using tightly focused femtosecond pulsed lasers (Figure 11d).^210–213^ Because the probability of two-photon absorption scales with the square of the local photon flux, radical generation is restricted to the focal volume of the beam. This nonlinearity produces a voxel that is significantly smaller than the diffraction-limited focal spot, enabling submicrometer, and, in optimized systems, sub-100 nm, feature sizes.

Resolution can be further improved through stimulated emission depletion (STED)-type strategies, in which a second, donut-shaped depletion beam suppresses excitation at the periphery of the focal volume, yielding printed features in the tens-of-nanometers regime.^214^ These methods provide unmatched spatial precision and remain the only additive manufacturing technologies capable of reliably producing architected polymer structures with true nanoscale features.

The primary limitation of 2PP is throughput. Because polymerization occurs voxel-by-voxel, construction of millimeter- or centimeter-scale objects requires significantly longer printing times than single-photon methods. Recent developments, including parallelization efforts, the emergence of (1 + 1)-photon processes and hybrid sequential/nonlinear exposure schemes, have begun to mitigate this limitation, but 2PP remains best suited to applications where resolution is paramount rather than manufacturing speed.^12^

#### 4.1.3 Volumetric 3D Printing

Volumetric 3D printing encompasses exposure schemes in which a three-dimensional light dose is delivered throughout the resin volume, enabling objects to be fabricated in a single operation rather than through sequential layer formation. The most established implementation is tomographic volumetric 3D printing, introduced by Taylor and co-workers under the names computed axial lithography (CAL) and tomographic volumetric additive manufacturing (VAM).^12,202,203,215^ In this approach, a cylindrical volume of photoresin is rotated, while it is irradiated with a sequence of dynamically evolving two-dimensional projections (Figure 11e). These projections represent filtered back-projections of the target geometry, analogous to the inverse of a medical CT scan. When superimposed in the rotating resin, they produce a spatially localized cumulative dose that exceeds the polymerization threshold only within the intended 3D region, solidifying the object in tens of seconds.^12^

By eliminating the need for layerwise recoating, tomographic volumetric printing avoids stair-stepping artifacts, supports highly isotropic geometries, and achieves resolutions comparable to conventional single-photon methods but with orders-of-magnitude faster build times. The hardware requirements are modest, a projector, rotation stage, and optical path, which has facilitated rapid dissemination of the technique across research laboratories. Nevertheless, application to polyester-based resins remains limited because tomographic printing requires formulations with high optical transparency, sufficient viscosity stability, and fast, threshold-dominated polymerization kinetics, criteria that conventional SLA or DLP polyester resins do not always satisfy.^12^

A related class of volumetric technologies is light-sheet 3D printing, in which polymerization is confined to the thin intersection of two orthogonally delivered light fields (Figure 11f).^216–218^ A first wavelength (λ_1_) excites the photoinitiator to a long-lived intermediate state that does not initiate polymerization; only upon absorption of a second wavelength (λ_2_) is a reactive species generated. Polymerization therefore occurs exclusively where the λ_1_ light-sheet and the λ_2_ projection overlap, enabling spatially selective curing at arbitrary positions within the vat without resin replenishment. This two-color, two-step excitation strategy, realized using either conventional photoinitiators with appropriate excited-state manifolds or photoswitch-based systems such as spiropyran–merocyanine—underpins both research-scale implementations and the recently commercialized xolography platform.^216–222^

Depending on the photoinitiator system, light-sheet methods achieve resolutions from ∼20 μm (xolography) down to the submicrometer regime when employing excited-triplet-state two-step initiators, while offering substantially higher printing speeds than nonlinear voxel-localized techniques. Despite these advantages, their use with polyester-based formulations is still sparse, primarily due to the stringent requirements for optical clarity, viscosity stability, and suitable two-wavelength photoinitiator systems, which remain commercially limited.

### 4.2 Evolution of Light-Based 3D Printing of Polyesters: From Feasibility to Functional Constructs

The progression of light-based 3D printing of polyesters spans more than two decades, moving from the earliest demonstrations that degradable polyester networks could be photocured into stable 3D objects toward increasingly sophisticated constructs designed for specific biological and technological functions. Initial reports established the fundamental feasibility of processing polyester-based photoresists in stereolithography and digital light processing, typically yielding simple architectures that nonetheless revealed how macromolecular structures govern degradability and curing behavior. Subsequent efforts adopted light-based 3D printing as a means to produce architected scaffolds with application-focused geometries, enabling controlled porosity, tissue specificity, and improved biological integration. Parallel developments introduced shape-memory and deployable polyester systems, while advances in two-photon polymerization and volumetric printing extended the accessible resolution and fabrication speed across several orders of magnitude. Most recent studies have incorporated additional chemical and functional capabilities, ranging from radioactivity to drug release, highlighting the expanding design space accessible when polyester chemistry is combined with modern light-based 3D printing techniques. The subsections below follow this trajectory, beginning with the first reports in which polyester photocuring was shown to be compatible with light-based additive manufacturing; all relevant studies are summarized in Table 1 (vide infra).

**Table 1:** State-of-the-Art of Light-Based 3D Printing of Polyesters

year	polymer	molar mass (g mol^–1^)	no.	functionality	printing technique	ref
2000	CL-TMC copolymer (0.5:0.5)	2200, 4300	2	coumarin	SLA	^177^
		2900, 8100, 12 000	3			
		5300, 13 000, 7400	4			

2002	CL-TMC copolymer (0.5:0.5)	2200	2	acrylate	SLA	^226^
	CL-TMC copolymer (0.5:0.5) with central block of PEG1000	3300	2			

2009	PCL–PTMC–PEG triblock copolymer	1050	2	methacrylate	2PP	^236^
2011	PCL	810, 1550, 2680, 4200, 6030	3	methacrylate	heat-assisted SLA	^227^
2012, 2015	PLA	1290	4	methacrylate	2PP	^237–239^
2016, 2017	PCL	10 000	2	methacrylate	heat-assisted SLA	^230,231^
2017	PCL	ND	2	acrylate	DLP	^232^
2018	PGS	ND	2	methacrylate	SLA	^240^

2018	PCL	530, 1250	2	acrylate	DLP, 2PP	^92^
		300, 900	3			

2019	PCL	530, 1250	2	acrylate	2PP	^114^
		300, 900	3			

2019, 2021	PCL	2990–3965	2	acrylate	DLP	^228,229^
	PCL-TMC copolymers (90:10, 75:25, 50:50, 25:75 and 10:90)	300, 900	3			

2020	PCL + GelMA	2000	4	methacrylate	DLP	^128^
2020	PLLA	423	3	methacrylate	DLP	^132^
2020, 2023	PCL–PLA copolymers (5:1, 5:1, 5:2, 6:2, 9:4)	1000, 2500, 4000, 6000, 10 000	2	acrylate	heat-assisted DLP	^143^
2021	PPG–PCL–PPG triblock copolymer	3000 g·mol^–1^ PCL, 4000, 6000, or 8000 g·mol^–1^ PPG	2	acrylate	DLP	^233^
2021	PCL	900	3	methacrylate	DLP	^100^

2021	PCL–PLA copolymer (7:3)	15000	4	methacrylate	DLP	^234^
	PCL–PLA copolymer (5:5)	600	2	methacrylate		

2021	PCL	9020	2	acrylate	2PP	^141^
		11300	6			

2022	PCL–PLA copolymer (1:1)	8700	4	methacrylate	DLP	^235^
2022	PCL	2000	2	acrylate, thiol–ene, thiol–yne	DLP	^145^

2023	PCL–PLA copolymers (2:6, 2:6, 2:6, 2:6, 2:8)	4360, 7340, 8780, 11390, 8430	2	acrylate	DLP, 2PP	^142^
	PCL–PLA copolymer (4:6)	1950, 11890	4			

2023	PCL	2300, 4700, 6400, 8700	2	thiol–ene	VP	^91^
2023	PCL	8000, 7900, 7700	2/3/4	thiol–ene	DLP	^90^
2023	poly(ether-ester)	monomer	NA	photoacid	DLP	^185^

2024	PCL	550	ND	acrylate	DLP	^247^
	PLA–PUA, CLOP blend	ND				

2024	PLA with lignin blend	ND	ND	acrylate	DLP	^124^
2024	PCL with CT contrast agent	2300, 4000	2	thiol–ene	VP	^147^
2024	PCL-allylPCL statistical and block copolymer	11 200 (statistical), 10 800 (block)	2	thiol–ene	DLP	^246^
2016, 2018, 2021, 2022, 2023, 2024	poly(diol citrate)	ND	ND	methacrylate	CLIP, DLP	^243,254,255^
2025	PCL with RGD	8000	3	thiol–ene	VP	^242^
2025	PCL	8000	3	thiol–ene	VP	^241^

Although methacrylate-functionalized PHB oligomers have been shown to photocure under UV irradiation, no PHA-derived network has yet been demonstrated as a standalone photoresist in stereolithography or related light-based 3D printing modalities.^223–225^

#### 4.2.1 From Feasibility to the First Degradable Polyester Constructs

The earliest demonstrations of light-based 3D printing of polyesters established that degradable aliphatic polyesters could be formulated into photoresists that withstand the optical, rheological, and kinetic constraints imposed by stereolithography and digital projection printing. These foundational studies revealed the boundary conditions under which polyester macromers can be photo-cross-linked into stable 3D structures, and in doing so, they defined the architectural and molecular features that later enabled more advanced constructs.

The earliest of these contributions came from Matsuda and co-workers in 2000, who prepared PCL–PTMC copolymers end-functionalized with coumarin, enabling single-component photo-cross-linking through [2 + 2] cycloaddition under SLA exposure (Figure 12a).^177^ Although the printed objects were architecturally simple, the study demonstrated several principles that remain central today: that degradable polyesters can be rendered photocurable without relying on acrylate chemistry, that multiarm architectures improve photocuring efficiency at fixed molar mass, and that light-based 3D printing can produce degradable scaffolds without the need for added photoinitiator. The follow-up study employing acrylated PCL–PTMC copolymers extended this feasibility window and introduced the notion that tailored polyester composition, here through PEG incorporation, modulates hydrolytic degradation and drug-release behavior in printed microneedles (Figure 12b).^226^

**Figure 12:**
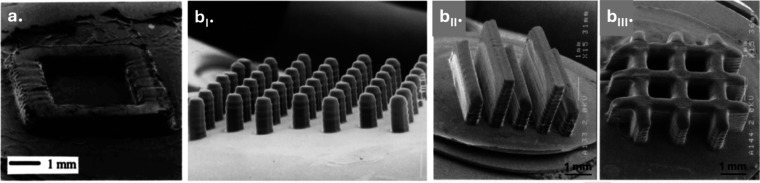
First polyester-based 3D prints (2000) using a photoresist based on a poly­(ε-caprolactone-*co*-trimethylene carbonate) copolymer, functionalized with (a) coumarin, and (b) acrylate, yielding microneedle-arrays (i), microbanks (ii), and microwells (iii), as reported by Matsuda et al. Reproduced with permission from refs 177, 226 . Copyright 2000 American Chemical Society (ref 177), and 2002 Wiley (ref 226), respectively.

Together, these early demonstrations established the fundamental design rules for polyester photoresists: precursor molar mass must balance functional-group density with suppressible crystallinity; multiarm or otherwise architecture-rich macromers substantially improve curing behavior; printability is highly sensitive to resin viscosity and photoreactivity; and degradability remains tightly linked to the underlying polyester chemistry, independent of the photochemical cross-linking strategy. These insights set the stage for subsequent developments in which polyester networks were no longer merely printable but increasingly architected, application-specific, mechanically tunable, and functionally integrated.

#### 4.2.2 Architected Polyester Structure toward Specific Applications

Once feasibility had been firmly established, research in light-based 3D printing of polyesters moved toward *purpose-driven architectural design*: constructs were no longer printed simply to demonstrate that polyester resins were compatible with a given modality but to address specific biomedical or mechanical requirements that demanded controlled porosity, spatial organization, and mechanically relevant geometries. This stage of development marked the transition from proof-of-concept networks to architected scaffolds whose utility stemmed from their geometry as much as from their chemistry.

A major driver behind this shift was the recognition that degradable polyesters, particularly PCL, PLA, PTMC, and their copolymers, possess mechanical and biological properties that align naturally with tissue-engineering paradigms. Porous scaffolds, lattices with tunable stiffness, and constructs with controlled Z-porosity became common targets for stereolithography and DLP formulations, and the literature began to emphasize the interdependence among resin viscosity, photoreactivity, and the complexity of the printed geometry.

The earliest examples of architected polyester structures emerged from heat-assisted SLA. In 2011, Elomaa et al. fabricated highly porous PCL networks with several-hundred-micrometer pore sizes using trifunctional PCL macromers printed above their melting point (Figure 13a).^227^ Operating the resin above *T*_m_ circumvented the viscosity and crystallization constraints that often limit polyester processing. The absence of shrinkage reported in this system underscored the interplay between precursor molar mass, thermal state, and cross-linking kinetics, an interplay that continues to define modern polyester formulations across printing modalities.

**Figure 13:**
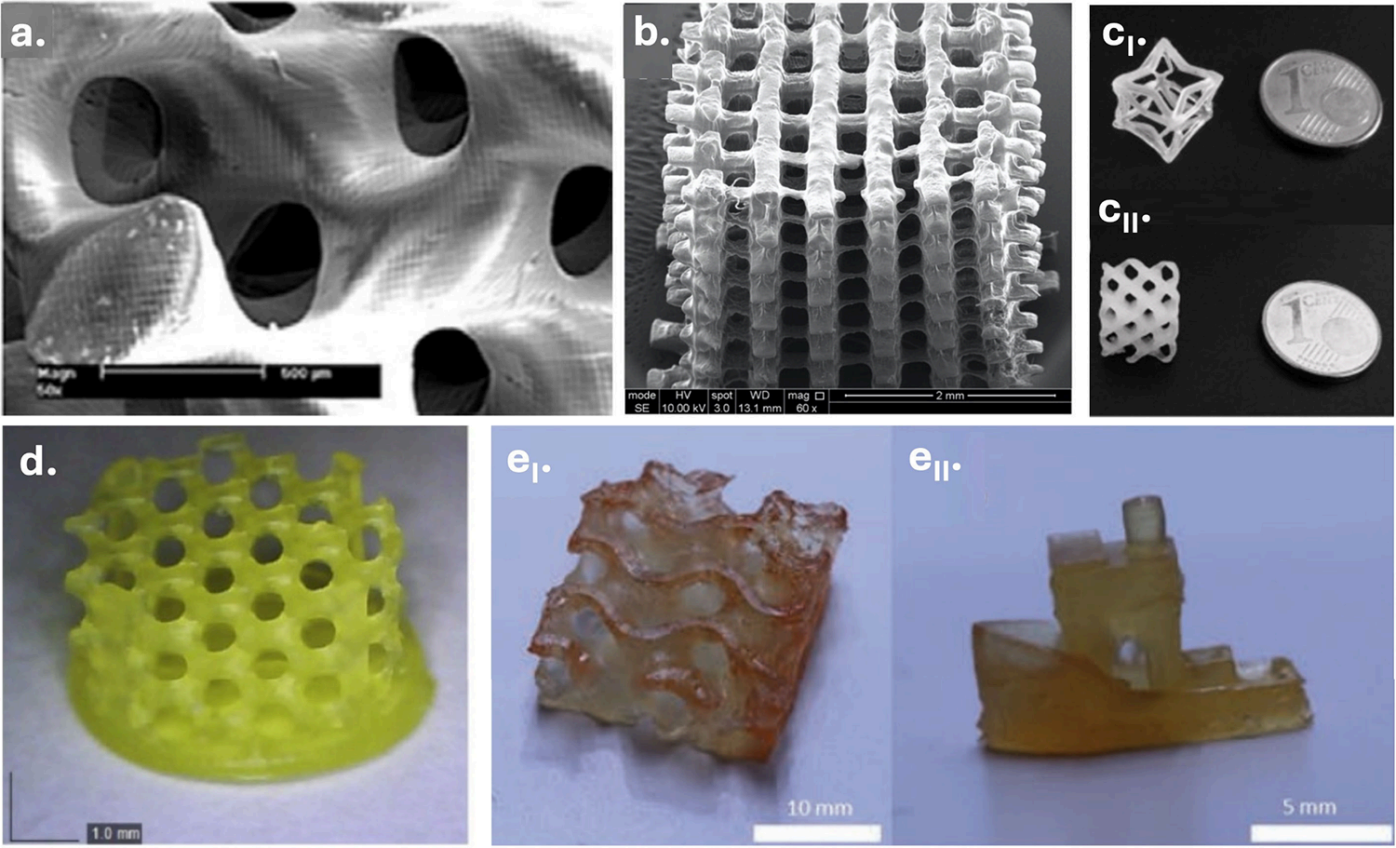
Architected polyester constructs produced by single-photon printing. (a) SLA of highly porous PCL structures by Elomaa et al,^227^ (b) DLP of three-arm methacrylated PCL by Field et al,^100^ (c) heat-assisted DLP of crystalline PCL–PTMC acrylate resins by Kuhnt et al,^228^ (d) DLP of methacrylated poly­(l-lactic acid) by Saed et al,^132^ (e) DLP of photo-cross-linkable PCL through acrylate, thiol–acrylate, and thiol–ene photo-cross-linking chemistry by Thijssen et al.^145^ Reproduced with permission from refs 227, 100, 228, 132, 145 . Copyright, respectively, 2011 Elsevier (ref 227), 2021 MDPI (ref 100), 2021 Elsevier (ref 228), 2020 Elsevier (ref 132), and 2022 Wiley (ref 145).

Subsequent work expanded architectural control using room-temperature DLP. Green et al. printed telechelic and trifunctional PCL macromers into sheet-like and moderately complex 3D architectures designed for retinal tissue interfaces.^92^ Although the constructs did not yet achieve a high geometric complexity, their cytocompatibility and successful implantation in vivo underscored the relevance of polyester structures as biomedical interfaces. The work also highlighted how architectural demands dictate precursor choice: only macromers with sufficiently low molar mass and low crystallinity yielded resins that could produce structurally coherent designs.

The first explicit demonstration of architected designs with reliable Z-porosity was reported by Field et al. using three-arm methacrylated PCL precursors (Figure 13b).^100^ These formulations produced scaffolds with true through-thickness porosity, enabling nutrient transport and cell infiltration in ways that earlier polyester constructs could not. Although the resulting networks remained mechanically limited due to chain-growth heterogeneity, the study clearly established the feasibility of building polyester architectures that respond to the biological requirement for open-pore, three-dimensional environments.

More advanced application-driven architectures emerged in 2019–2021 with the development of polyester lattices optimized for mechanical functionality. Wang et al. demonstrated that PCL–PLA copolymers, functionalized with acrylates via a convergent end-cap strategy, with tailored molar mass and composition could be processed by DLP into mechanically tunable constructs whose stiffness and extensibility are directly matched to tissue-specific needs, enabling soft, elastic matrices for regenerative scaffolding.^143^ In a complementary approach, Kuhnt et al. developed polyester urethane acrylate resins based on PCL–PTMC copolymers, using composition variation and end-group functionalization to tune crystallinity and mechanical response. Their follow-up work further enabled heat-assisted DLP of high-crystallinity resins by integrating a temperature-controlled build stage, demonstrating how process adjustments can extend the range of compatible polyester formulations (Figure 13c).^228,229^ Saed et al. extended this principle to PLA-based gyroid scaffolds for hard-tissue engineering, showing how curing depth, dye concentration, and exposure time together define pore fidelity and compressive performance in architected lattices (Figure 13d).^132^

As these studies accumulated, the role of the photopolymerization mechanism became increasingly intertwined with architectural capability. The work by Thijssen et al. on acrylate, acrylate–thiol, thiol–ene, and thiol–yne PCL systems illustrated how network topology and cross-link density fundamentally governs the mechanical robustness of architected polyester lattices (Figure 13e).^145^ Thiol–ene networks, in particular, enabled the fabrication of highly elastic and mechanically resilient architectures with feature sizes as small as 100 μm, capabilities that earlier acrylate-based constructs could not achieve. Importantly, these designs were not simply thicker, smoother versions of fragile polyester networks; they represented a deliberate alignment of architectural freedom with a step-growth photochemical mechanism that produces homogeneous strand-length distributions. Architected structures thus became a direct expression of underlying network design principles rather than a merely geometric output.

Collectively, these contributions reveal a clear conceptual trajectory: as soon as polyester photopolymerization moved beyond feasibility, architectural design rapidly became the central variable through which degradable, biocompatible, and mechanically appropriate structures could be engineered. Whether targeting porous nerve-guidance conduits, retinal interfaces, gyroid lattices for bone tissue engineering, or soft elastic networks for load-bearing constructs, the literature in this period demonstrates that architectural programmability is not orthogonal to resin chemistry, it is enabled and constrained by it. By the time shape-memory systems (section 4.2.3) and high-resolution or volumetric modalities (section 4.2.4) emerged, the groundwork for application-driven architecture design had already been firmly established.

#### 4.2.3 Shape-Memory and Deployable Polyester Constructs

As polyester photoresins matured from early feasibility toward application-specific architectures, a distinct subfield emerged that leveraged the semicrystalline nature or glass transition of polyesters to enable shape-memory and deployable constructs. These systems exploit the reversible crystallite formation in PCL, PLA, or PCL-containing (co)­polymers and/or *T*_g_ transitions to program temporary shapes that recover upon heating. Light-based 3D printing proved uniquely suited for this purpose: it allowed spatially patterned cross-link densities, controlled pore geometries, and programmable segment lengths, parameters that directly govern shape-fixity and recovery ratios.

The first light-based demonstrations of polyester shape-memory behavior were reported by Zarek et al. in 2016–2017 (Figure 14a).^230,231^ Using heat-assisted SLA, they processed methacrylated PCL oligomers (*M*_n_ ≈ 10000 g·mol^–1^) into highly crystalline networks whose thermal transitions enabled large, reversible deformations. Printing above the melting point suppressed crystallization during fabrication, allowing uniform curing; upon cooling, the networks crystallized to yield high storage moduli and excellent shape-fixity. These studies illustrated that crystallinity can be a functional advantage after printing and that light-based fabrication enables intricate, monolithic shape-memory constructs beyond what mold-based methods can achieve (see section 4.3.2 for a detailed discussion of crystallization during formulation). Later variants integrated electrical conductivity or electronic triggering, emphasizing that deployability, rather than simple softness or print fidelity, was the central design driver.

**Figure 14:**
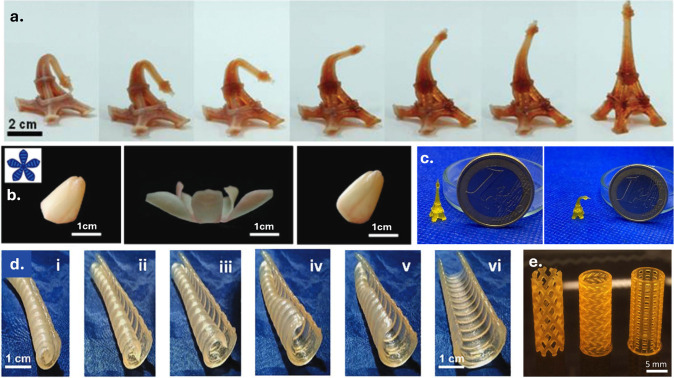
Shape-memory and deployable polyester constructs. (a) SLA-printed shape memory PCL networks by Zarek et al,^230^ (b) patterned swelling and shape-change behavior in PCL diacrylate films by Huang et al,^232^ (c) shape memory constructs printed from poly­(ε-caprolactone-lactic acid) copolymers by Delaey et al.,^142^ (d) DLP-printed shape-memory tracheal stents from poly­(propylene glycol)/PCL triblock copolymers by Maity et al.,^233^ (e) DLP of poly­(d,l-lactide-*co*-ε-caprolactone) networks with different molar masses by Paunović et al.^234^ Reproduced with permission from refs 230, 232, 142, 233, 234 . Copyright, respectively 2016, 2017, 2023, 2021 Wiley (refs 230, 232, 142, 233 ), and 2021 AAAS (ref 234.).

Subsequent developments expanded the chemical and architectural scope of shape-memory polyesters. In 2017, Huang et al. used digital grayscale illumination to modulate local cross-link densities in PCL diacrylate films, producing patterned swelling and shape-change behavior without layerwise fabrication (Figure 14b).^232^ Although the approach produced relatively simple, nonfreestanding geometries, it demonstrated that spatial control of the network topology, even within a uniform polyester formulation, can program complex shape transformations, providing a direct chemical mechanism for 4D printing.

More recently, Delaey et al. broadened the scope of shape-memory polyesters to include multiarm PLA–PCL urethane-based architectures printable via both DLP and 2PP (Figure 14c).^142^ These resins combined tunable crystallinity with network elasticity, allowing shape-memory behavior even at low moduli (<0.8 MPa).

A more targeted biomedical implementation emerged in 2021 with the work of Maity et al., who printed shape-memory tracheal stents from PPG–PCL triblock copolymers functionalized with acrylates (Figure 14d).^233^ By tuning the relative block lengths (PPG segments, 2 000–8 000 g·mol^–1^; PCL segment, 2 000 g·mol^–1^), the authors adjusted both the shape-memory transition temperature and the mechanical compliance of the constructs. These stents demonstrated reversible deployment and could also deliver small-molecule drugs, underscoring how shape-memory behavior enables minimally invasive insertion followed by in situ expansion, an application uniquely dependent on both the chemistry and the printed geometry.

Photothermal activation introduced an additional dimension of deployability. In 2022, Paunović et al. incorporated gold nanorods into methacrylated PLA–PCL copolymers to generate near-infrared-responsive shape-memory constructs (Figure 14e).^234,235^ This approach eliminated the need for global heating: irradiation at biologically benign wavelengths triggered localized heating of the embedded nanorods, enabling selective recovery of preprogrammed shapes. Such constructs illustrate how shape-memory can be coupled to optical control, bridging the gap between structural programmability and functional responsiveness.

Throughout these developments, the unifying theme is that polyester chemistry inherently enables shape-memory behavior and light-based 3D printing provides the architectural and spatial control necessary to exploit it. From early heat-assisted SLA and grayscale exposure to photothermal actuation and triblock design, these systems demonstrate that deployability is not merely an added property, it is a direct consequence of controlled crystallization, network architecture, and spatially structured photopolymerization. As later sections (sections 4.2.4 and 4.2.5) illustrate, these principles would ultimately intersect with high-resolution printing and functional augmentation, but shape-memory constructs represent the first class of polyester architectures in which form and function were engineered in tandem.

#### 4.2.4 High-Resolution and Ultrafast Printing of Polyesters

As polyester formulations matured beyond early feasibility and application-specific scaffolds, researchers began exploring printing modalities that extend the capabilities of conventional SLA and DLP. Two directions dominated this transition: (i) the pursuit of submicrometer feature sizes enabled by nonlinear absorption processes and (ii) the development of volumetric approaches capable of fabricating centimeter-scale geometries in seconds. Both trajectories required substantial rethinking of the resin architecture, photoinitiator selection, and optical clarity, placing fundamentally new constraints on polyester chemistry.

The first indication that biodegradable polyesters could be patterned at micrometer-scale resolution emerged in 2009, when Claeyssens and co-workers demonstrated two-photon polymerization of a PCL–PTMC–PEG triblock copolymer (Figure 15a).^236^ Using a benzophenone-based initiator, they achieved features as small as ∼4 μm, an important proof-of-principle that polyester backbones, despite their intrinsic crystallinity and hydrophobicity, could be integrated into nonlinear voxel-based fabrication. Follow-up studies by the same group extended this to methacrylated four-arm PLA, producing structures that supported neuroblastoma and Schwann cell cultures (Figure 15b–d).^237–239^ They also showed that appropriate macromer design, short chain length, multiarm architecture, and high functional-group density, produces polyester networks that can be reliably polymerized both in 2PP and in SLA.^240^ The architectural precision afforded by 2PP enabled scaffold designs impossible to achieve by layer-based methods, but printing speeds remained slow and resin requirements remained stringent, particularly with respect to light transport and initiator efficiency in the focal volume.

**Figure 15:**
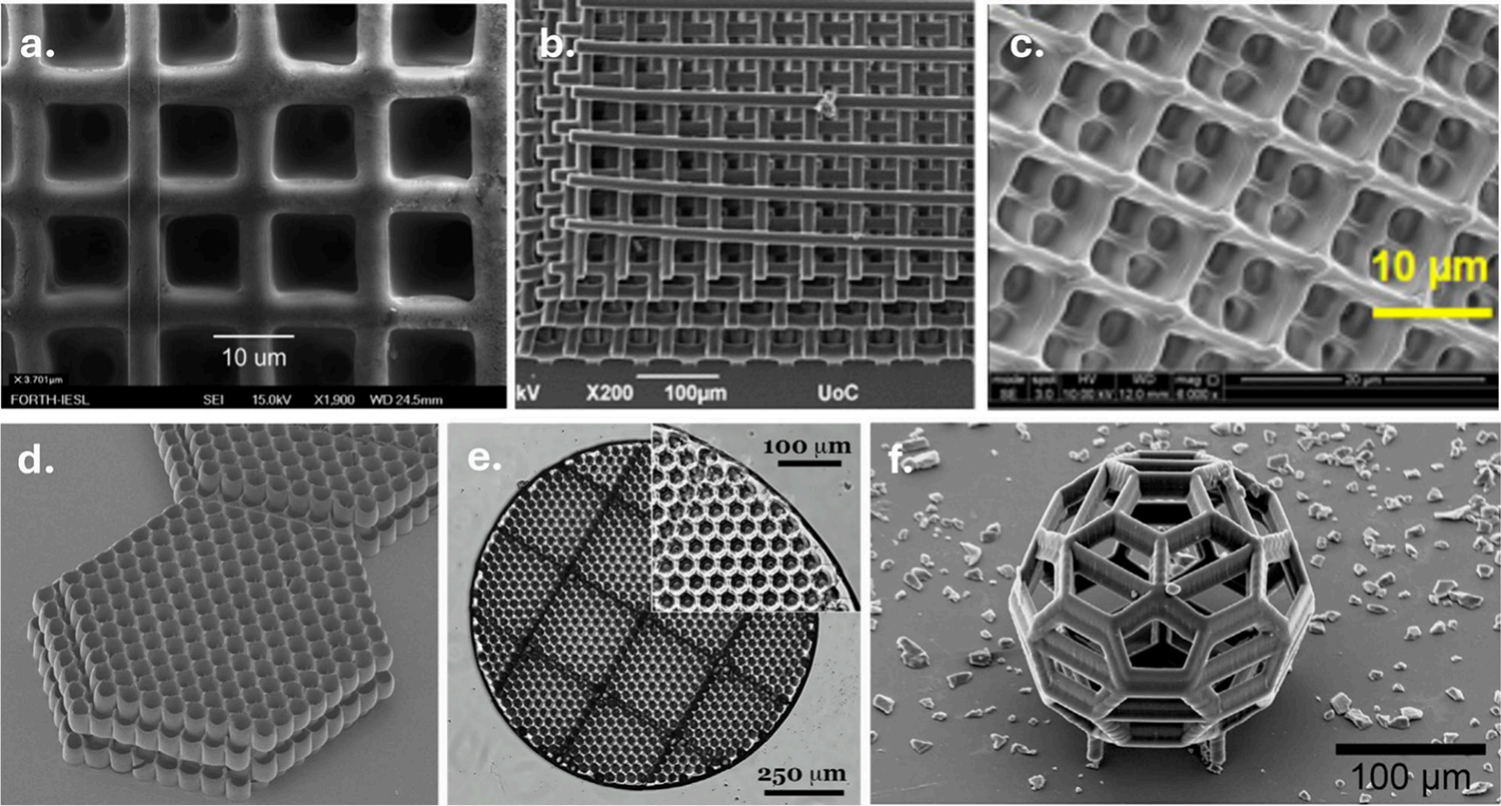
High-resolution two-photon printing of polyester-based constructs. (a) First 2PP-fabricated microstructures from PCL–PTMC–PEG triblock copolymers by Claeyssens et al,^236^ (b–d) four-arm PLA and PGSm printed through 2PP,^237–239^ (e) acrylated PCL scaffolds for retinal cell growth produced by 2PP by Thompson et al,^114^ (f) 2PP-construct of acrylated PCL with the highest resolution to date by Arslan et al.^141^ Reproduced with permission from refs 236–239, 114, 141 . Copyright, respectively, 2009 American Chemical Society (ref 236), 2015 Elsevier (ref 237), 2015 Springer Nature (ref 238), 2012 IOP Publishing (ref 239), 2019 Elsevier (ref 114), and 2020 Elsevier (ref 141).

This evolution was also exemplified by follow-up work to Green et al.’s DLP-printed PCL constructs for retinal scaffolds, which relied on low-*M*_n_, low-crystallinity macromers for structural fidelity and cytocompatibility (section 4.2.2).^92^ Thompson et al. later adapted these same macromers for two-photon polymerization by optimizing photoinitiator type, laser power, and scanning speed, while maintaining a low precursor molar mass to ensure high voxel reactivity (Figure 15e).^114^ The resulting microarchitectures supported retinal cell growth in vitro and in vivo, demonstrating that careful resin tuning enables polyester-based constructs to transition across printing modalities.

Advances in initiator chemistry further expanded the resolution frontier. In 2021, Arslan et al. used telechelic PCL functionalized with either one or three acrylates per chain end (*M*_n_ ≈ 10000 g·mol^–1^) to achieve features as small as 143 ± 18 nm, the smallest reported for biodegradable polymers to date (Figure 15f).^141^ Although these networks exhibited brittle failure and moderate strength, the work established that polyester-based resins are compatible with the most demanding high-resolution photofabrication techniques. In parallel, Delaey et al. showed that shape-memory PLA–PCL urethane systems could also be processed via 2PP, demonstrating that multimodal printability (DLP + 2PP) is achievable when the resin’s photochemistry and segmental mobility are carefully designed.^142^

In contrast to the resolution-driven trajectory of 2PP, the development of tomographic volumetric printing (CAL/VAM) introduced a paradigm focused on speed, isotropy, and elimination of layer artifacts. The first polyester-based volumetric photoresist was reported only recently, in 2023, when Thijssen et al. introduced a thiol–ene PCL system optimized for threshold-dominated cure under tomographic dose delivery (Figure 16a).^91,147^ The resin combined high optical transparency, rapid step-growth polymerization kinetics, and tunable *M*_*c*_ values (2300–8700 g·mol^–1^), enabling the fabrication of cubic and octahedron lattices with feature sizes down to 100 μm in tens of seconds. The homogeneous network topology of the thiol–ene reaction improved toughness relative to acrylate analogues, producing extensible networks (elongation at break up to 630%) capable of withstanding the stresses generated during rapid volumetric curing. This platform was further expanded by Quaak et al., who synthesized linear, trifunctional, and tetra-functional PCL architectures bearing allyl groups for thiol–ene photo-cross-linking (Figure 16b).^90^

**Figure 16:**
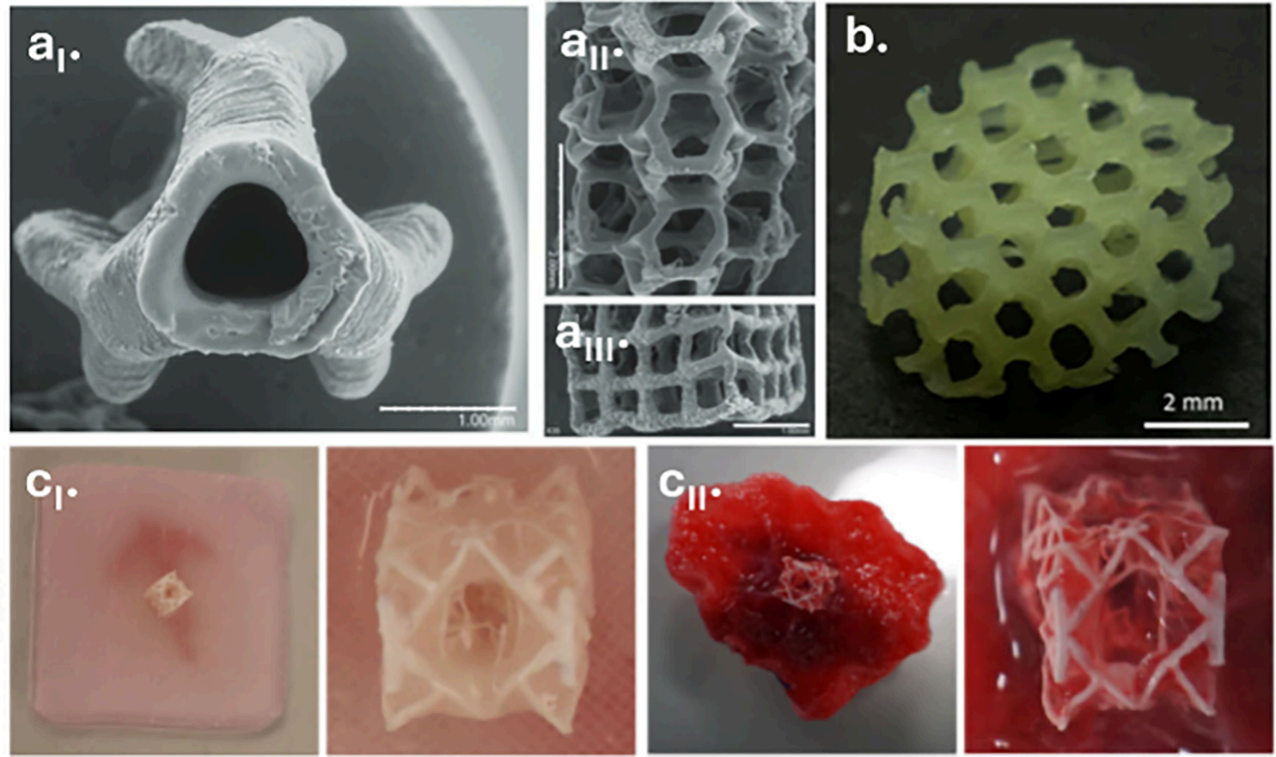
Volumetric and architected-DLP printing of polyester networks. (a) First report on volumetric 3D printing of polyesters using thiol–ene photo-cross-linkable PCL of (i) a vascular tree, (ii) a strut-like octahedron, and (iii) a cubic lattice with strut sizes of 100 μm by Thijssen et al,^91^ (b) DLP-printed gyroid structure of thiol–ene photo-cross-linkable PCL with varying architecture by Quaak et al,^90^ (c) reinforced cardiac tissue patch from VP-PCL metamaterials by Jones et al.^241^ Reproduced with permission from refs 91, 90, 241 . Copyright, respectively, 2023 Wiley (ref 91), 2023 Royal Society of Chemistry (ref 90), and 2025 Wiley (ref 241).

Building on these structurally focused VP resins, Jones et al. designed a library of anisotropic truss-based PCL metamaterials with myocardium-matched stiffness using a graph-based generative modeling framework and fabricated them by volumetric printing (Figure 16c).^241^ By integrating these VP-PCL metamaterials with a melt-electrowritten mesh and a cardiomyocyte-laden fibrin hydrogel, they created a reinforced cardiac tissue patch that withstood intraventricular pressures and prevented bleeding in an acute large-animal defect model, illustrating how a polyester-based VP can be extended to clinically relevant, multimaterial implants.

Together, these studies mark a critical expansion of the polyester 3D printing landscape. Two-photon polymerization demonstrated that polyesters can reach nanoscale architectural control, enabling scaffold designs guided by cellular length scales. Volumetric printing, on the other hand, demonstrated that complex polyester geometries can be fabricated in the time frame of seconds rather than hours without compromising structural fidelity.

#### 4.2.5 Polyester Constructs with Integrated Chemical and Functional Capabilities

As polyester-based photoresists matured in both print fidelity and mechanical performance, researchers increasingly turned toward expanding their functionality beyond structural support. This transition reflects a broader shift in the field: rather than viewing polyesters solely as degradable scaffolds, they are now engineered as platforms that integrate biological, mechanical, radiological, or therapeutic functions directly into printed object. These efforts required not only new monomer and macromer designs but also careful balancing of photoreactivity and network formation to preserve printability across SLA, DLP, and, in more recent cases, volumetric modalities.

One of the earliest demonstrations of this trend was reported by Elomaa et al., who blended methacrylated gelatin (GelMA) with methacrylated PCL to produce DLP-printable constructs with inherent cell adhesiveness (Figure 17a).^128^ Although the printed geometries remained relatively simple, the study showed that hydrophilic, hydrophobic polyester hybrids could improve biological integration without compromising processability. This approach established a framework for combining bioactive or hydrophilic components with hydrophobic polyester segments to produce resins with heterogeneous, but functionally beneficial microenvironments.

**Figure 17:**
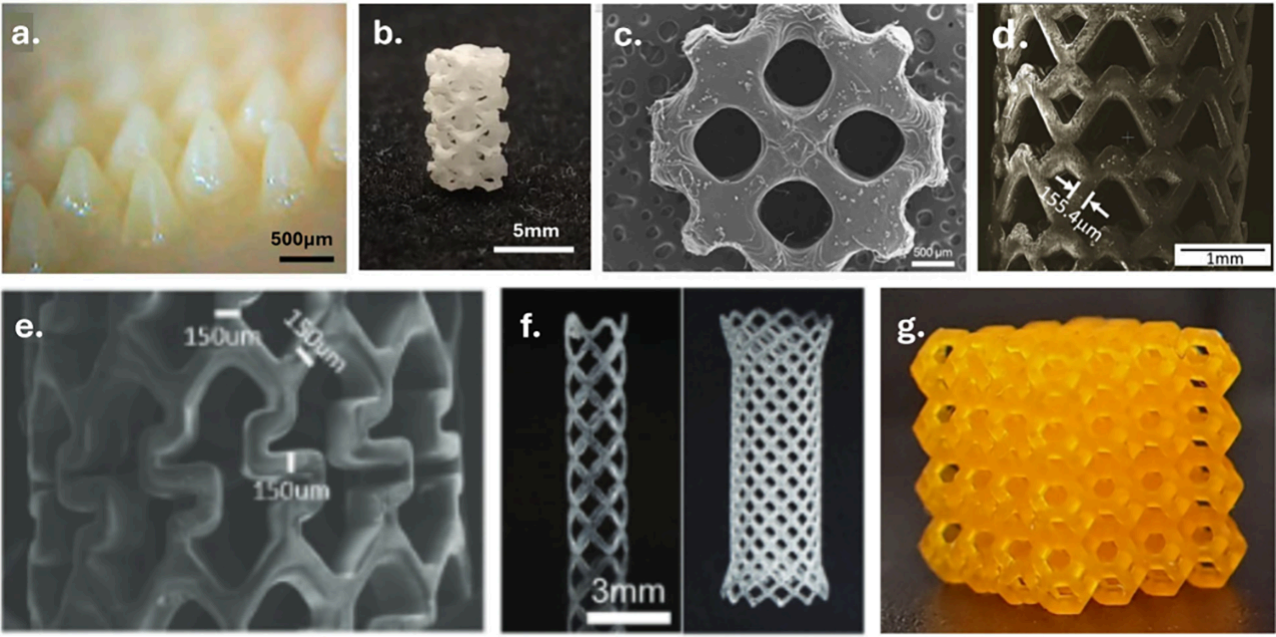
Polyester constructs with integrated chemical and functional capabilities. (a) DLP-printed construct from blended methacrylated gelatin with methacrylated PCL by Elomaa et al,^128^ (b) RGD-incorporated PCL construct through volumetric 3D printing by Thijssen et al,^242^ (c) SEM images of volumetrically printed triply periodic minimal surface (I-WP) of thiol–ene cross-linkable PCL with CT contrast agent AATIPA by Kolouchova et al,^147^ (d,e) CLIP-printed poly­(diol citrate) stents with antioxidant properties,^243,244^ (f) CLIP-printed poly­(diol citrate) vascular grafts by Ding et al,^245^ (g) DLP-printed copolymer-construct of ε-caprolactone and allyl-ε-caprolactone by Torres et al.^246^ Reproduced with permission from refs 128, 242, 147, 243–246 . Copyright, respectively, 2020, 2025, 2025 Elsevier (refs 128, 242, 244 ), 2016, 2018, 2022 Wiley (refs 147, 243, 245 ), and 2024 American Chemical Society (ref 246).

More recently, Thijssen et al. addressed the polarity mismatch between hydrophilic cell-adhesive peptides and hydrophobic PCL matrices by formulating a dimethylformamide-based thiol–ene resin in which a cysteine-terminated RGD peptide and alkene-functionalized PCL are codissolved and covalently integrated during volumetric 3D printing (Figure 17b).^242^ By carefully balancing peptide solubility, attenuation, radical inhibition, and viscosity, the resin maintained a sharp solidification threshold and enabled tomographic fabrication of porous, cell-adhesive PCL architectures in tens of seconds at peptide loadings up to ∼0.3 wt %, supporting integrin-dependent adhesion and spreading of multiple cell types. This work demonstrates that bioactivity can be embedded throughout hydrophobic polyester VP networks during printing, rather than being restricted to postprint surface modification.

The integration of radiopaque moieties into polyester-based networks represents another direction for functional augmentation. Kolouchova et al. covalently incorporated 5-acrylamido-2,4,6-triiodoisophthalic acid (AATIPA) into alkene-functionalized PCL networks, enabling nondestructive CT monitoring of scaffold degradation (Figure 17c).^147^ The resulting materials supported volumetric printing of triply periodic minimal surfaces, the first demonstration of CT-visible polyester constructs fabricated through VP. Their study showed that the stringent requirements of volumetric printing, minimal light scattering, rapid yet uniform polymerization, and high viscosity stability, can be met even when additional functional components are integrated into the polyester backbone.

The possibility of embedding therapeutic agents directly into polyester resins was also been explored. Adhami et al. incorporated clopidogrel into PLA–PUA/PCL-based DLP resins to create antithrombogenic vascular grafts with tunable drug loading (5–20 wt %).^247^ Despite the complexity introduced by the drug molecules, affecting viscosity, radical reactivity, and phase compatibility, the printed grafts closely matched the mechanical properties of native vessels and remained structurally stable under physiological loading.

A complementary strategy for augmenting performance involves blending polyesters with functional fillers. Guessasma et al. introduced lignin (5–30 wt %) into acrylated PLA resins to enhance mechanical toughness and sustainability.^124^ Although high lignin loadings led to phase separation and brittleness, low loadings improved mechanical properties while retaining print fidelity, underscoring how filler–matrix interactions must be optimized to match the photopolymerization and optical requirements of DLP resins.

Furthermore, from 2016 to 2024, Cheng Sun and co-workers reported a series of CLIP-based 3D printing studies of methacrylated poly­(diol citrate), primarily focusing on the development of cardiovascular stents with various implemented functionalities (Figure 17d,e). Poly­(diol citrate) is reported to have antioxidant properties, and the developed stents exhibited self-expanding behavior.^243,244^ They also demonstrated CLIP of a composite of methacrylated poly­(diol citrate) and PLA to reinforce the 3D printed materials.^248^ Additionally, they developed radiopaque stents by incorporating iodixanol into the poly­(diol citrate)-based photoresist and created drug-releasing 3D printed parts (Figure 17f).^245,249,250^ In parallel, Oliveira et al. reported methacrylated poly­(diol citrate) formulations incorporating nitric oxide (NO)-releasing or NO-modulating functionalities for 3D printed biodegradable vascular constructs, achieving antibacterial activity and tunable degradation.

Finally, resin architectures have been adapted not only for added chemical functionality but also for thermomechanical versatility. Torres et al. synthesized copolymers of ε-caprolactone and allyl-ε-caprolactone in both statistical and block configurations to modulate crystallinity, enabling a single chemical platform to produce amorphous, semicrystalline, and shape-memory constructs upon DLP printing (Figure 17g).^246^ By tuning block architecture rather than introducing new reactive sites, they demonstrated that functional performance can also be programmed through sequence design—an increasingly important tool in developing multifunctional polyester-based photoresists.

### 4.3 Resin Considerations for Light-Based 3D Printing of Polyesters

The formulation of a polyester-based photoresin represents the final step linking the macromolecular structure to printing performance. Whereas section 2 established how polymerization mechanisms define the intrinsic architecture of polyester precursors, and section 3 outlined how photoreactive groups are introduced and cross-linked into networks, it is the resin formulation that ultimately determines whether these precursors can be converted into reliable, high-fidelity printed objects. Resin composition governs how light propagates through the medium, how rapidly reactive groups encounter one another, and how the material behaves during the printing process, whether sequential (SLA, DLP), continuous (CLIP), nonlinear (2PP), or volumetric (VP).

This subsection therefore examines the chemical and physical principles that control resin behavior specifically for polyester systems. The discussion aims to move beyond empirical formulation recipes toward the broader conceptual patterns that consistently determine success: the density and spatial distribution of photoreactive functionality, the suppression or management of crystallization during printing, and the way these factors propagate into the physicochemical properties of 3D printed polyesters. Together, these insights highlight that resin formulation is not a peripheral consideration but the point at which polyester chemistry, photopolymerization kinetics, and hardware-specific constraints converge.

#### 4.3.1 Functional-Group Density and Architectural Design As the Foundational Resin Constraint

The starting point for any polyester photoresin is the density and spatial organization of photoreactive groups that can participate in network formation.^90,100,141,142,145,177^ Unlike conventional acrylate or epoxy resins, where monomeric or oligomeric species naturally carry high concentrations of vinyl or epoxide functionalities, polyester-based systems inherit their reactive sites from the precursor itself.^92,100,128^ As a result, functional-group density is not introduced during formulation but is predetermined by the precursor’s molar mass, functionality, and architecture, all of which are fixed upstream during synthesis and functionalization.^90,92,100,141,143,145,177,246^ These inherited parameters dictate the statistical likelihood that reactive sites will meet and form a network under the time, dose, and diffusion constraints of a given printing modality. Functional-group density and architectural precision therefore represent the most fundamental resin-level constraints for polyester-based 3D printing.^108^

For end-functionalized precursors, the molar mass of the polyester sets the concentration of reactive ends per unit volume: as the *M*_n_ increases, the number of photoreactive groups decreases. In practice, all polyester photoresins converge on a relatively narrow precursor *M*_n_ range, typically 1–6 kg·mol^–1^, where terminal functionalities remain sufficiently concentrated to sustain rapid curing while preserving a useful mechanical plateau after cross-linking.^91,92,100,142,143,177,227^

Polymer architecture provides a second orthogonal lever to control functional-group density at the resin level. Telechelic diols produce two reactive sites per chain, whereas three- and four-arm star architectures provide three or four end-groups at comparable per-chain *M*_n_.^90,100,142,227^ Alternatively, single-end-functional precursors can carry multiple reactive groups at one terminus, increasing functional density without changing backbone architecture.^141^ Because the network connectivity at gelation depends on the ratio of functionality to molar mass, star architectures enable gelation at lower conversion and thus require lower printing light doses.^90^ Beyond end-group multiplicity, the spatial placement of reactive groups, whether solely at chain termini or distributed along the backbone, modulates the reactivity and the ultimate properties of the printed polyester. Linear α,ω-functional polyesters produce well-defined molar mass between cross-links (*M*_*c*_), whereas statistical or block copolymers bearing in-chain reactive sites (e.g., allyl-bearing CL units) produce less homogeneous network topologies and *M*_*c*_.^246^

Crucially, these architectural decisions determine not only the achievable cure kinetics but also the physicochemical properties of the ultimate printed object.^90,92,144,145,251^ Networks derived from low-*M*_n_, high-functionality precursors yield high cross-link densities, rapid gelation, and sharp cure fronts, a combination well-suited for all light-based 3D printing techniques. However, such networks tend to be brittle.^100,141,145^ Conversely, higher-*M*_n_ precursors (>6–7 kg·mol^–1^) or precursors with lower functional group density typically require higher light doses and result in incomplete conversion, yet they maintain the properties of the initial polyester backbone to a larger extend.^90,91,142,143,177,246^ Thus, there exists a trade-off for the polyester resin design that balances printability and the ultimate physicochemical properties.

Across the chronology of polyester 3D printing, these principles repeatedly explain which systems succeed. Early coumarin- and acrylate-functional PCL-TMC resins printed reliably because their *M*_n_ (2 kg·mol^–1^) and trifunctional architectures ensured dense, accessible functional groups.^177,226^ Two-photon printed PCL and PLA systems used low-*M*_n_ four-arm precursors to achieve high voxel reactivity.^237–239^ Modern VP resins similarly rely on low-*M*_n_ telechelic or multiarm PCL because they concentrate reactive groups sufficiently to enable volumetric gelation at moderate doses.^91,241^ Even complex formulations incorporating fillers, drugs, or radiopaque monomers only succeed when the underlying functional-group density is sufficiently high to form a continuous network around these additives.^147,242^

Functional-group density and architectural design therefore govern not only whether a polyester can be printed but also how sharply it resolves features and how the resulting network distributes stress, crystallizes, and degrades. Most resin-level trade-offs, between print fidelity and toughness, between crystallinity suppression and mechanical strength, or between dilution and cure speed, ultimately trace back to how much reactive functionality is embedded in each polymer chain and how that functionality is architecturally organized.

#### 4.3.2 Crystallization as the Central Formulation Boundary

Crystallization is an intrinsic property of many aliphatic polyesters, such as PCL, PLA, and related polyesters, and arises whenever the polyester chemical structure, chain mobility, concentration, and temperature combine to allow nucleation.^227,230,252^ During resin preparation, this crystallization can be fully suppressed by dissolving the polyester in a reactive monomer, solvent, or diluent, or by working above *T*_m_; however, suppression is not elimination.^90–92,141,227,230,231,245,246^ Any increase in concentration (e.g., through solvent evaporation), any local cooling, or any composition gradient can trigger crystallite formation. Once crystallites nucleate, they scatter light, disrupt dose delivery, and destroying print fidelity.^92,141^

Thus, the practical concentration window of a polyester in a photoresin is not set by solubility alone but by how close that composition lies to the crystallization boundary on the time and temperature scales of printing. Star-shaped, branched, or architecturally disrupted polyesters expand this printable window because they suppress crystallization relative to linear analogues.^90,100,141,142,227^ Copolymerization with amorphous blocks, PDLLA, PEG, PTMC, or random CL-*co*-LA sequences, achieves the same effect.^229,233,235,236,249^

Crystallization links directly back to the polyester molar mass and architecture. Increasing *M*_n_ raises the enthalpic drive for crystallization and narrows the miscibility window, while reducing *M*_n_ or employing multiarm structures broadens it.^90–92,100,141^ Reactive diluents, solvents, and/or monomer-rich formulations reduce crystallization risk but simultaneously lower functional-group density. Heat-assisted printing avoids this contradiction by working above *T*_m_, allowing high-*M*_n_, crystalline polyesters to flow without solvent/diluents.

Finally, crystallization, though detrimental during printing, becomes a powerful design tool after curing. Once the solvent is removed or the network is allowed to cool into its semicrystalline regime, crystallites contribute stiffness, toughness, and shape-memory behavior, improving mechanical performance far beyond what the amorphous network alone could provide.^142,230,232,233^

#### 4.3.3 Reactive Diluents Diminish the Polyester Contribution

Reactive diluents are widely used in polyester photoresins because polyester backbones tend to slow photopolymerization.^100,128,143,253^ Their low local functional-group density, large interhandle spacing, and flexible aliphatic segments reduce propagation rates and cross-link density compared to small-molecule acrylate formulations. Reactive diluents therefore became a practical means to increase reactivity, sharpen exposure windows, and improve printability under typical SLA and DLP conditions.^228,233,235^

Diluents influence curing through two main effects. First, they affect viscosity, allowing for rapid resin replenishment in layer-based modalities and limiting sedimentation in volumetric printing. Second, small-molecule acrylates introduce a large number of reactive sites per unit volume, accelerating kinetics and raising cross-link density.^143^ These improvements in printability, however, come at the expense of the properties that motivate the use of polyester backbones in the first place, as increasing the diluent fraction progressively reduces the polyester contribution to crystallinity, segmental organization, mechanical response, and hydrolytic degradation. High diluent loadings ultimately produce networks whose identity is governed by the diluent-rich matrix rather than by the polyester.^100,143,228^

Taken together, reactive diluents improve printability by compensating for the inherently slow kinetics and reduced functional density of the polyester macromers. Yet they simultaneously diminish the structural and chemical advantages that distinguish polyesters. Effective formulation therefore requires using only the minimum amount of reactive diluent needed for kinetic compatibility with the printing modality, while preserving the crystallinity, degradation behavior, and mechanical identity encoded in the polyester backbone.^233^

The mechanical, thermal, and degradation behavior of printed polyester architectures cannot be inferred from monomer structure or backbone identity alone. Final performance arises from a translation across three levels: polyester synthesis defines precursor molar mass, architecture, and crystallization tendency; functionalization and photochemistry determine how those precursors can be cross-linked; and resin formulation matches the developed system to the respective light-based 3D printing modality. The properties of a printed construct therefore reflect how the precursor structure, functional-group density, crystallization control, and dilution strategy combine during curing.

## 5 Conclusions and Future Perspectives

Light-based 3D printing of polyesters has progressed from early feasibility demonstrations to a mature and rapidly diversifying field in which molecular design, photochemistry, and fabrication strategies increasingly operate as an integrated framework. The advances synthesized in this review, spanning polyester synthesis, photochemical network formation, and resin formulation, have enabled high-resolution architectures, mechanically robust networks, and the introduction of selected functional capabilities across multiple printing modalities. Yet, despite this progress, several scientific and technological challenges now define the next stage of development and collectively outline a broad opportunity landscape for light-based 3D printing of polyesters.

A central challenge remains: the gap between the properties of thermoplastic polyesters and those achievable with current photoresins. High printability demands low molar mass and high densities of reactive groups, whereas high mechanical performance in polyesters is typically rooted in long chain segments, semicrystallinity, and well-defined phase organization. Reconciling these conflicting requirements, achieving rapid, high-resolution photopolymerization while preserving the desirable attributes of thermoplastic polyesters, represents a defining bottleneck for the field. Progress in this direction will require new approaches to embedding long-range molecular features within rapidly curing networks as well as new strategies for reintroducing crystallinity or segmental organization after printing.

Closely linked to this challenge is a fundamental gap in understanding how the structural features of a polyester precursor translate into the behavior of the cross-linked network. Although the influence of precursor molar mass, architecture, crystallinity, and end-group placement on thermoplastic polyesters is well established, their translation into the mechanical, thermal, and degradation performance of the printed network remains difficult to predict. Establishing quantitative relationships between precursor design, network topology, crystallization behavior, and degradation kinetics will be essential for moving from empirical resin development toward rationally engineered photoresins.

Addressing these structure–performance relationships will be accelerated by expanding the photochemical toolbox available for network formation. Most polyester-based photoresins rely on radical reactions that introduce nondegradable linkages and limit both recyclability and network homogeneity. The development of nonradical photochemistries, including photoacid- or photobase-triggered reactions, light-gated ring-opening pathways, reversible deprotection strategies, and rROP-inspired cross-linking, presents a promising direction for generating networks that are fully degradable, compositionally homogeneous, and better aligned with the chemical logic of polyester backbones. These alternatives may ultimately enable the creation of networks in which the defining characteristics of the underlying polyester are retained rather than overwritten during curing.

Beyond single-material systems, orthogonal photochemistry will be essential for enabling spatially heterogeneous and multimaterial constructs. Multiwavelength and multimechanism systems hold the potential to integrate soft, stiff, bioactive, or degradable domains within a single platform, capabilities that become particularly powerful in volumetric 3D printing, where true overprinting and embedded structures are possible. Such approaches open a path toward scaffolds with localized biological cues, devices with spatially programmed mechanics, and materials capable of dynamic or reconfigurable behavior.

Expanding material capabilities will, in turn, broaden the application space of light-based 3D printing of polyesters. Although biomedical engineering has been the dominant driver of the field, significant opportunities remain in soft robotics, programmable mechanical systems, microfluidics, environmental sensing, and sustainable manufacturing. Realizing the potential of polyester-based photoresins in these domains will rely on the ability to create networks that combine rapid, high-resolution processability with tailored mechanical and chemical performance, further underlining the importance of material innovations at the molecular and photochemical level.

Finally, meeting these challenges will require predictive tools that link polymer synthesis, photochemistry, curing behavior, network topology, and long-term degradation. Integrating kinetic modeling, light transport simulations, and machine-learning-assisted resin design offers an opportunity to reduce empirical trial-and-error and to accelerate the development of next-generation polyester resins. Coupling these computational approaches with high-throughput experimental workflows will be key to navigating the vast chemical and formulation space inherent to polyester-based photoprinting.

In summary, the future of light-based 3D printing of polyesters will be shaped by advances at the intersection of polymer synthesis, photochemistry, and 3D printing. Bridging the thermoplastic–photoresin performance gap, enabling fully degradable and orthogonally addressable networks, expanding application domains, and integrating sustainable design principles collectively define the next phase of progress. As these challenges are addressed, polyester photoprinting is poised to evolve from a predominantly biomedical platform into a general materials-design strategy with an impact across multiple technological sectors.

## Abbreviations

3D: three-dimensional
4D: four-dimensional
AI: artificial intelligence
UV: ultraviolet
NIR: near-infrared
ROP: ring-opening polymerization
ROCOP: ring-opening copolymerization
rROP: radical ring-opening polymerization
SLA: stereolithography
DLP: digital light processing
CLIP: continuous liquid interface processing
2PP: two-photon polymerization
VP: volumetric 3D printing
CAL: computed axial lithography
M_c_: molar mass between cross-links
PCL: poly­(ε-caprolactone)
PLA: poly­(lactic acid)
PGA: poly­(glycolic acid)
PPF: poly­(propylene fumarate)
PGS: poly­(glycerol sebacate)
PET: poly­(ethylene terephthalate)
PBT: poly­(butylene terephthalate)
PBS: poly­(butylene succinate)
PTMC: poly­(trimethylene carbonate)
PEG: poly­(ethylene glycol)
PPG: poly­(propylene glycol)
PLGA: poly­(lactide-*co*-glycidyl methacrylate)
PTMP: pentaerythritol tetrakis­(3-mercaptopropionate)
PUA: polyurethane acrylate
GelMA: gelatin methacryloyl
AATIPA: 5-acrylamido-2,4,6-triiodoisophthalic acid
RGD: Arg-Gly-Asp
